# Pharmacological Treatments for Congenital Myasthenic Syndromes Caused by *COLQ* Mutations

**DOI:** 10.2174/1570159X21666230126145652

**Published:** 2023-05-18

**Authors:** Shuai Shao, Guanzhong Shi, Fang-Fang Bi, Kun Huang

**Affiliations:** 1 Department of Neurology, Xiangya Hospital, Central South University, Changsha, Hunan Province, China;; 2 Xiangya School of Medicine, Central South University, Changsha, Hunan province, China;; 3 National Clinical Research Center for Geriatric Disorders, Xiangya Hospital, Central South University, Changsha, Hunan Province, China

**Keywords:** Congenital myasthenic syndrome, CMS, *COLQ*, β-adrenergic agonist, AChEIs, mutation

## Abstract

**Background:**

Congenital myasthenic syndromes (CMS) refer to a series of inherited disorders caused by defects in various proteins. Mutation in the collagen-like tail subunit of asymmetric acetylcholinesterase (*COLQ*) is the second-most common cause of CMS. However, data on pharmacological treatments are limited.

**Objective:**

In this study, we reviewed related reports to determine the most appropriate pharmacological strategy for CMS caused by *COLQ* mutations. A literature review and meta-analysis were also performed. PubMed, MEDLINE, Web of Science, and Cochrane Library databases were searched to identify studies published in English before July 22, 2022.

**Results:**

A total of 42 studies including 164 patients with CMS due to 72 different *COLQ* mutations were selected for evaluation. Most studies were case reports, and none were randomized clinical trials. Our meta-analysis revealed evidence that β-adrenergic agonists, including salbutamol and ephedrine, can be used as first-line pharmacological treatments for CMS patients with *COLQ* mutations, as 98.7% of patients (74/75) treated with β-adrenergic agonists showed positive effects. In addition, AChEIs should be avoided in CMS patients with *COLQ* mutations, as 90.5% (105/116) of patients treated with AChEIs showed either no or negative effects.

**Conclusion:**

(1) β-adrenergic agonist therapy is the first pharmacological strategy for treating CMS with *COLQ* mutations. (2) AChEIs should be avoided in patients with CMS with *COLQ* mutations.

## 1 INTRODUCTION

**Fig. (1):**
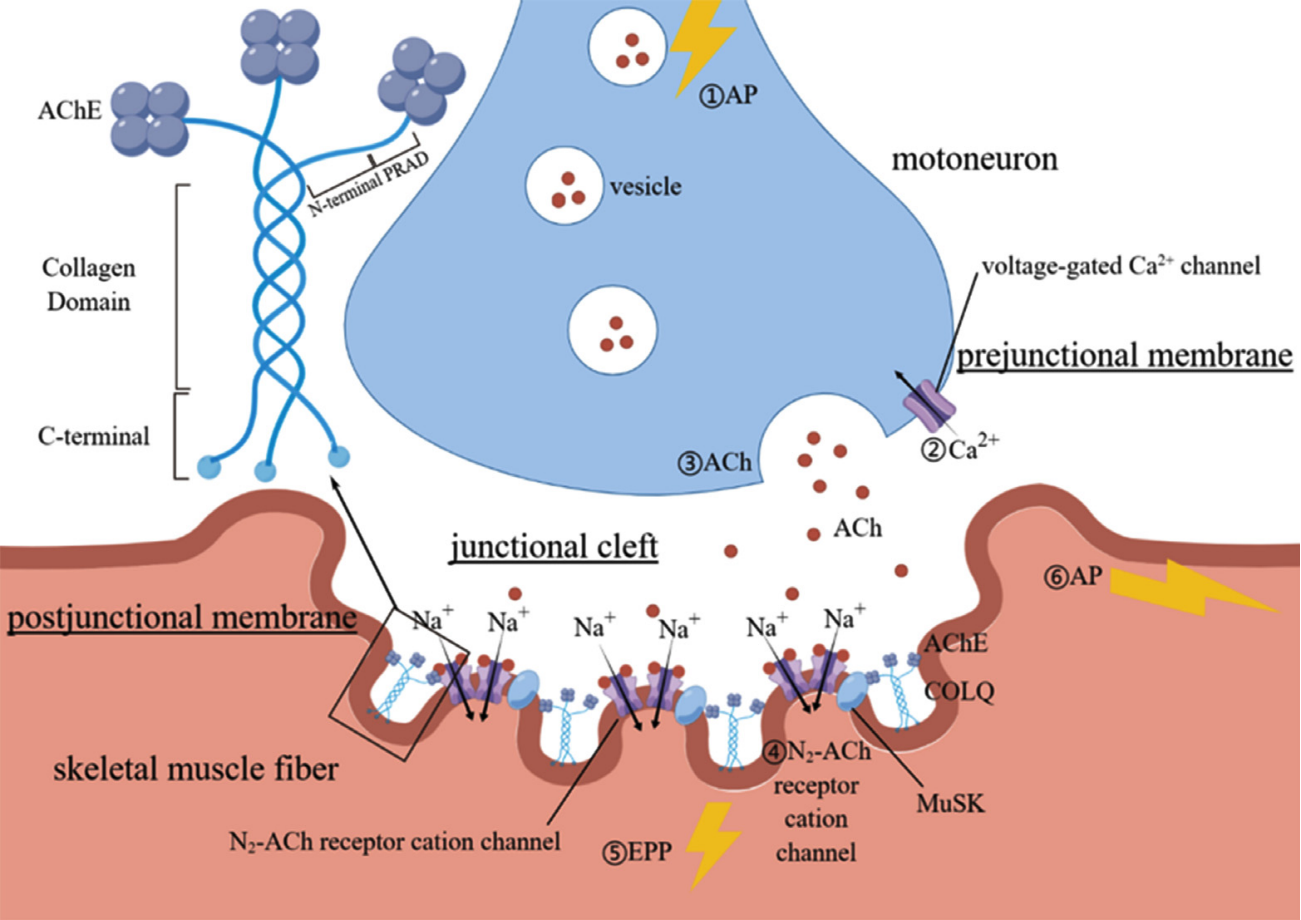
Structure of neuromuscular junction and components of COLQ. When the action potential (AP) of motoneurons activates voltage-gated Ca^2+^ channels on the prejunctional membrane, acetylcholine (ACh) is released quantally into junctional cleft. N_2_-ACh receptor cation channels on postjunctional membrane are activated, inducing Na^+^ inflow and K^+^ outflow. The end-plate potential (EPP) is mainly formed by Na^+^ inflow and spreads along the postjunctional membrane, forming the AP of skeletal muscle fiber. COLQ is crucial for the anchoring of AChE and the postjunctional membrane. It consists of an N-terminal proline-rich region attachment domain (PRAD), a triple helical homo-trimeric collagen-like tail subunit structure and a C-terminal region enriched in charged residues and cysteines. The figure was drawnby Figdraw.

The neuromuscular junction (NMJ) is a type of synaptic connection bridging motoneurons and skeletal muscle fibers that converts the electrical impulses of motoneurons into action potentials of muscle fibers [1]. When electrical impulses of motoneurons activate voltage-gated Ca^2+^ channels on the prejunctional membrane, acetylcholine (ACh) is released quantally into the junctional cleft. N_2_-ACh receptor cation channels on the postjunctional membrane are activated, enabling skeletal muscle fiber contraction. ACh is cleared within several milliseconds of release, mainly by acetylcholinesterase (AChE) located on the postjunctional membrane (Fig. **1**). The collagen-like tail subunit of asymmetric AChE (COLQ) is crucial for anchoring AChE and the postjunctional membrane [2].

Congenital myasthenic syndromes (CMS) include a series of inherited disorders caused by defects in different proteins [3-5]. CMS may be caused by any abnormality in the highly complex processes of neuromuscular transmission (NMT), involving the synthesis and vesicle packaging of ACh, influx to the terminus of calcium ions regulated by the opening of ion channels, subsequent activation of a complex leading to exocytosis of ACh-filled vesicles, and transition and postsynaptic response to ACh. NMT is likely to release more transmitters than required to remain effective under various physiological conditions and stresses. This ability is called “safety factor”, which is compromised in CMS [1].

The prevalence of CMS in the total population is approximately 0.18 per 100,000 in Spain [6] and southern Brazil [7]. Among children, the prevalence of CMS is approximately 0.92 to 2.22 cases per 100,000 (0.92 per 100,000 children under 18 years old in UK [8] and 2.22 per 100,000 children under 19 years old in Slovenia [9]). Mutation of the *COLQ* gene located on the short arm of chromosome 3 p24.2 is one of the most frequent causes of CMS [10-13] and is the second most common cause of CMS [14]. The prevalence of CMS caused by mutations in *COLQ* is approximately 0.25 per million total population [6].

Significant research attention has been dedicated to developing CMS treatments. AChE inhibitors (AChEIs), such as pyridostigmine, enhance the effect of ACh quantum release by inhibiting decomposition [3]. 3,4-diaminopyridine (3,4-DAP) increases the number of ACh quanta by blocking voltage-gated potassium channels [15]. Long-lived open-channel blockers of the acetylcholine receptor (AChR) ion channel, including fluoxetine and quinidine, relieve cationic overloading of the postjunctional membrane by limiting the duration of prolonged synaptic currents. β-adrenergic receptor agonists (BAs), such as salbutamol and ephedrine, have also been reported to mitigate some patients” symptoms [3]. Although some previous studies have reviewed treatment targets and efficacy for CMS [16], no studies have reported a systematic review and meta-analysis of data on treatments for CMS patients with *COLQ* mutations.

COLQ attaches the globular catalytic subunits of AChE to the synaptic basal lamina, thus anchoring and accumulating AChE in the extracellular matrix [3]. AChE consists of an N-terminal proline-rich region attachment domain (PRAD), a triple-helical homotrimeric collagen-like tail subunit structure, and a C-terminal region enriched in charged residues and cysteines (Fig. **1**). The PRAD connects to the globular catalytic subunits, and the C-terminal region helps grapple to the basal lamina and initiates triple helix assembly while the collagen-like tail connects the two [3, 12, 17, 18]. COLQ is anchored to the NMJ by a C-terminal domain and heparan sulfate proteoglycan binding domain in the triple helical domain [17-20]. In addition to anchoring and linking, COLQ has been shown to control the aggregation of N_2_-ACh receptor cation channels and gene expression of synapses through its interaction with muscle-specific receptor tyrosine kinase (MuSK), thus demonstrating a synaptic regulation function [21].

More than 70 mutations in *COLQ* have been identified thus far, and their locations and effects are listed in Fig. (**2**) and Supplementary Table **S1**. Frameshift or nonsense mutations can truncate the protein distal to PRAD [17, 22], whereas missense substitutions in the C-terminal region may hamper grappling to the basal lamina [23]. These mutations in *COLQ* may lead to a deficiency in endplate AChE and thus insufficient ACh elimination ability. ACh in the synaptic space will only be eliminated by diffusion or by other esterases, such as butyrylcholinesterase, instead of the decomposition effect of endplate AChE. As a result, the interaction between ACh and AChR is more frequent and long-lasting [24]. Excessive exposure of AChR at the postsynaptic membrane to ACh may block NMT due to receptor desensitization, exhibiting a mechanism similar to that of anticholinesterase drug poisoning [25, 26].

Initially, two phenotypes of CMS with *COLQ* mutations were reported. One is the “classical” phenotype, which onsets at birth, induces moderate to very severe weakness, and does not respond to AChEIs [27-29]. The other phenotype is onset at 6-7 years, that induces mild weakness with slow or no progression of symptoms, and does not respond to AChEIs [12, 29, 30]. As more cases of CMS with *COLQ* mutations have been identified, two more phenotypes have been recognized: later onset with graver symptoms or earlier onset with milder symptoms [22].

Different therapies have been applied for treating CMS in clinical settings. However, due to the interactions of various mechanisms, the same drug may have varying or even opposite effects on different CMS subtypes, which makes it necessary to analyze pharmacological treatments based on disease subtypes. Here, we reviewed related reports and attempted to determine the most appropriate pharmacological strategy for CMS caused by *COLQ* mutations.

## 2 METHODS

### 2.1 Search Strategy

The literature search was restricted to articles published in English. The search strategy was modified from that reported in a previous study [31-34]. We searched the PubMed (1966-2022), MEDLINE (1950-2022), Web of Science (1864-2022), and Cochrane Library (2022) databases using the keywords “*COLQ* mutations,” “*COLQ,*” or “congenital myasthenic syndrome, *COLQ*”. The most recent search was performed on July 22, 2022. In addition, a manual search was performed to identify references in the obtained studies to identify other possible studies. The preferred reporting items for systematic reviews and meta-analyses (PRISMA) [35] chart for the search strategy is shown in Fig. (**3**). No randomized clinical trials (RCTs) were identified, and only observational case reports or studies were included in our analysis. The studies were read thoroughly to assess their eligibility for inclusion in the meta-analysis.

### 2.2 Inclusion and Exclusion Criteria

Case reports and studies were included if they fulfilled the following criteria: (1) English-language articles; (2) patients with *COLQ* mutations with no restriction regarding their age, sex, ethnicity, and treatment; and (3) genetic tests confirmed the *COLQ* mutation regardless of the details of the mutations. The exclusion criteria were as follows: (1) articles not reporting pharmacological treatment(s) and (2) articles that did not mention the treatment effect of a pharmacological therapy.

### 2.3 Data Extraction

The following data were extracted directly from the articles: clinical information, gene mutations, pharmacological treatments, and follow-up data. Clinical data including sex, age at symptom onset, and age at the time of the case study were reported. If more than one patient had been reported in different studies or by the same group, the participants in the studies were compared. Data that appeared more than once were excluded from the analysis.

### 2.4 Quality Assessment of Individual Studies

The quality of individual studies was assessed using the recently published “Tool for evaluating the methodological quality of case reports and case series” proposed by Murad *et al.* [36] based on the previous criteria of the Pierson, Bradford Hills, and Newcastle-Ottawa scale. Each study was independently evaluated according to four domains (selection, ascertainment, causality, and reporting) to yield an overall assessment (Supplementary Table **S2**). The quality assessment included eight leading exploratory questions with a binary response (yes/no) to determine whether the items suggested the presence of bias.

### 2.5 Statistical Analysis

Data analysis was performed using Statistical Package for Social Sciences (SPSS Inc., Chicago, IL, USA) version 25. Statistical differences were estimated using the Kruskal-Wallis test or one-way analysis of variance followed by the Bonferroni post-hoc test. The analysis of the relationship between sex and treatment effect was estimated using the Mann-Whitney test (two groups). Simple linear regression was used to analyze the relationship between age of onset and treatment effect. Statistical significance was set at *p* < 0.05.

## 3 RESULTS

### 3.1 Search and Selection Results

After searching, 42 studies meeting our inclusion and exclusion criteria were selected, including 164 patients with CMS due to 72 different *COLQ* mutations [6, 12-14, 17, 19, 22, 27-30, 37-61]. Most selected studies were case reports, and none were RCTs. The information for each patient is listed in Supplementary Table **S3**, including geographical settings, mutation, onset age, related risk factors, treatment, and effects. Six different pharmacological strategies were used, including AChE inhibitors (AChEIs), a type of drug that inhibits ACh hydrolysis; β-adrenergic receptor agonists (BAs); 3,4-DAP, a drug that increases ACh release from the nerve terminal by blocking voltage-gated potassium channels (VGKCs); fluoxetine (FLX); glucocorticosteroids (GCs); and other drugs (Table **1**). Some patients underwent more than one kind of treatment. Because the effects of different strategies are described as negative, partial, positive, or remarkable, we categorized the treatment effect into four types: (-) no effect, negative or ambiguous response; (+) partial, incomplete, moderate, modest/mild effect, or initial positive response followed by ineffectiveness; (++) beneficial, positive, or clear effect; (+++) remarkable, dramatic, or satisfying effect.

### 3.2 Pharmacological Treatment Effects in Patients with CMS due to *COLQ* Mutations

**Fig. (4):**
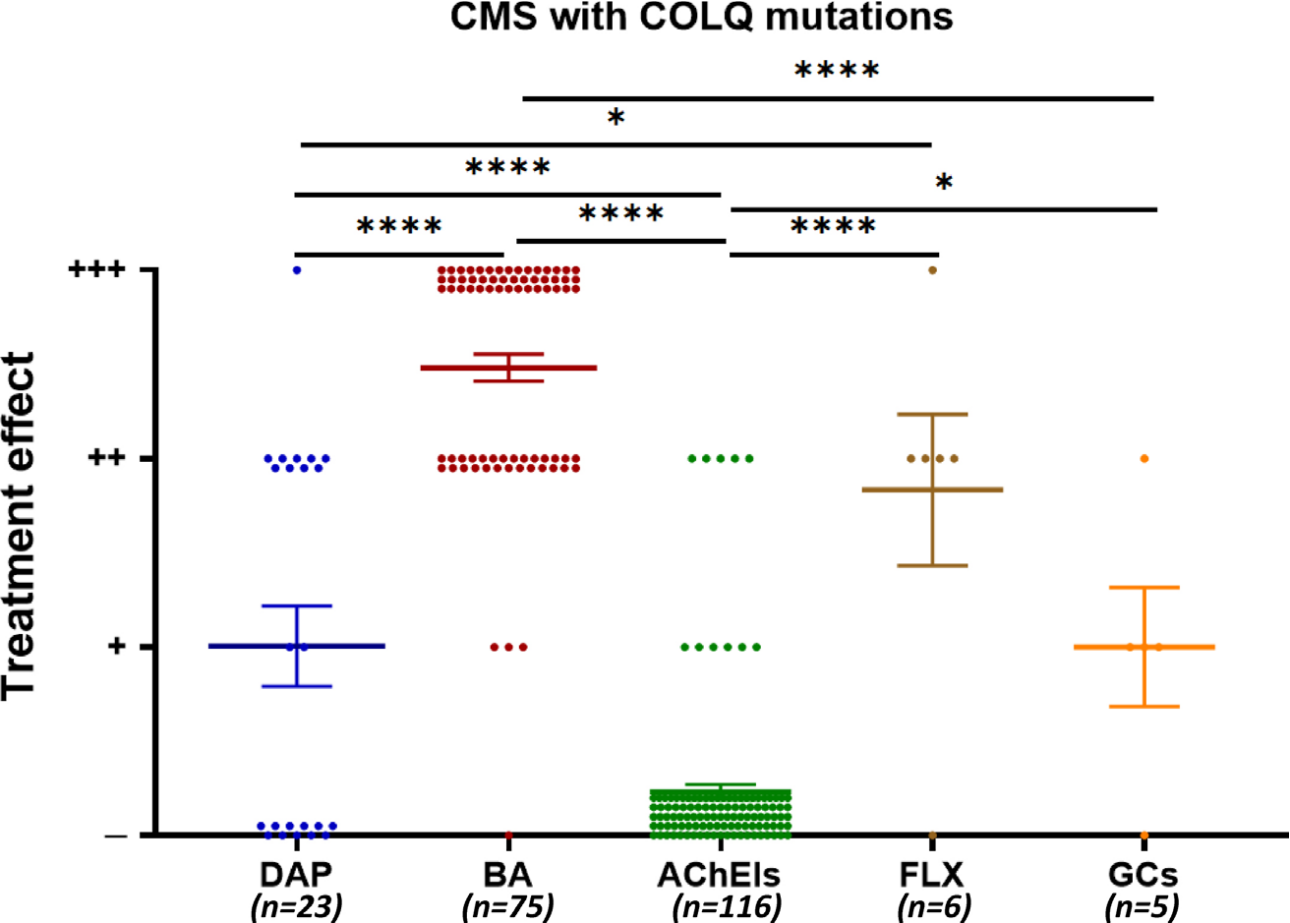
Pharmacological treatment effects in all CMS patients with *COLQ* mutations. Treatment effects were categorized into four types: (-) no effect, negative or ambiguous response; (+) partial, incomplete, moderate, modest/mild effect or denotes initial positive response followed by ineffectiveness and/or worsening; (++) beneficial, positive, or clear effect; (+++) remarkable, dramatic, or satisfying effect. DAP, 3,4-diaminopyridine; BA, β2-adrenergic receptor agonist; AChEI, acetylcholinesterase inhibitor; FLX, fluoxetine; GC, glucocorticoid. Each point represents one patient. Some patients underwent more than one kind of treatment. Mean and SEM are indicated. *p < 0.05 and ****p < 0.0001 by the Kruskal-Wallis test.

The selected studies reported on a total of 164 CMS patients diagnosed with *COLQ* mutations and 6 pharmacological treatment strategies. Additionally, four different treatment strategies were categorized as “other” because of their limited number. Among the five different strategies, β-adrenergic receptor agonists showed better effects than 3,4-DAP, AChEIs, and glucocorticosteroids (Fig. **4**).

Four different treatment strategies were categorized as “other” because of their limited number. Therefore, the group of others was not included in the statistical analysis. Their treatment effects are shown in Supplementary Table **S3**. The β-adrenergic receptor agonists showed the best treatment effect, benefiting 74 of the 75 patients treated (98.7%) (Fig. **4**). In addition, AChEIs showed no effect on 105 of the 116 patients treated (90.5%) (Fig. **4**).

### 3.3 Influence of Different Phenotypes on Treatment Effect

CMS patients with *COLQ* mutations were first divided into two phenotypes: the “classical” phenotype with early onset at birth and severe symptoms [27-29] and after-birth onset with mild symptoms [12, 29, 30]. Two more phenotypes showing later onset with severe symptoms or early onset with mild symptoms have since been recognized [22]. For our meta-analysis, we divided the patients into four categories: (1) “classic” phenotype: patients with severe symptoms and onset at birth (within 30 days after birth); (2) “early and mild” phenotypes: patients with slight to mild symptoms and onset at birth; (3) “late and severe” phenotypes: patients with severe symptoms and onset after birth (30 days after birth); (4) “late and mild” phenotypes: patients with slight to mild symptoms and onset after birth.

**Fig. (5):**
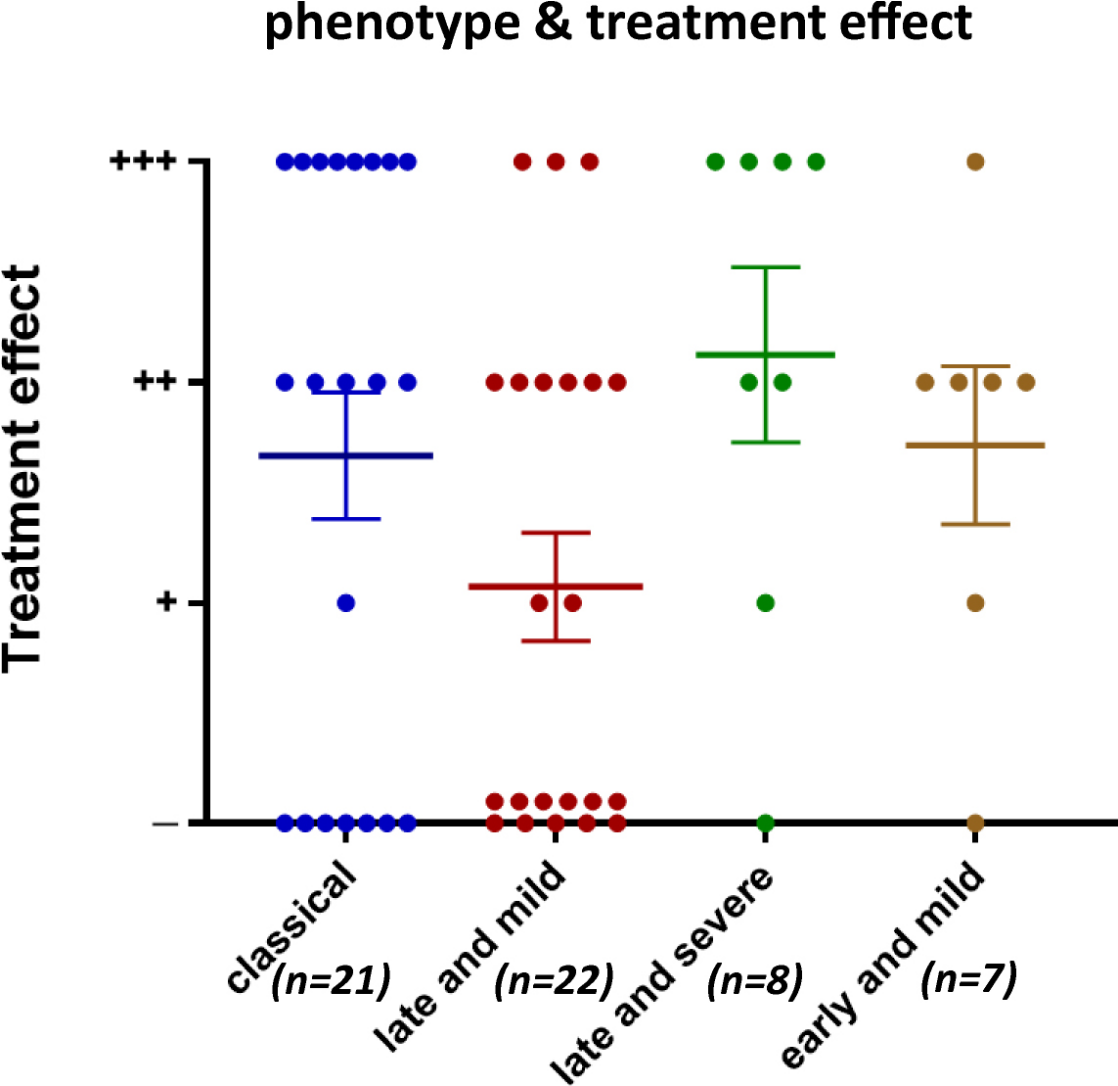
Treatment effects in CMS patients with *COLQ* mutations of different phenotypes. Treatment effects were categorized as noted in Fig. **4**. Each point represents one patient. Mean and SEM are indicated. No significant difference was found by the Kruskal-Wallis test (*p* > 0.05).

Data were evaluated according to phenotype and treatment effect where possible. Among the patients whose phenotypes were reported, 21 had the “classical” phenotype, 22 had the “late and mild” phenotype, 8 had the “late and severe” phenotype, and 7 had the “early and mild” phenotype. No significant difference in the treatment effect was found between the different phenotypes (Fig. **5**).

### 3.4 Treatment Effect According to Phenotype

**Fig. (6):**
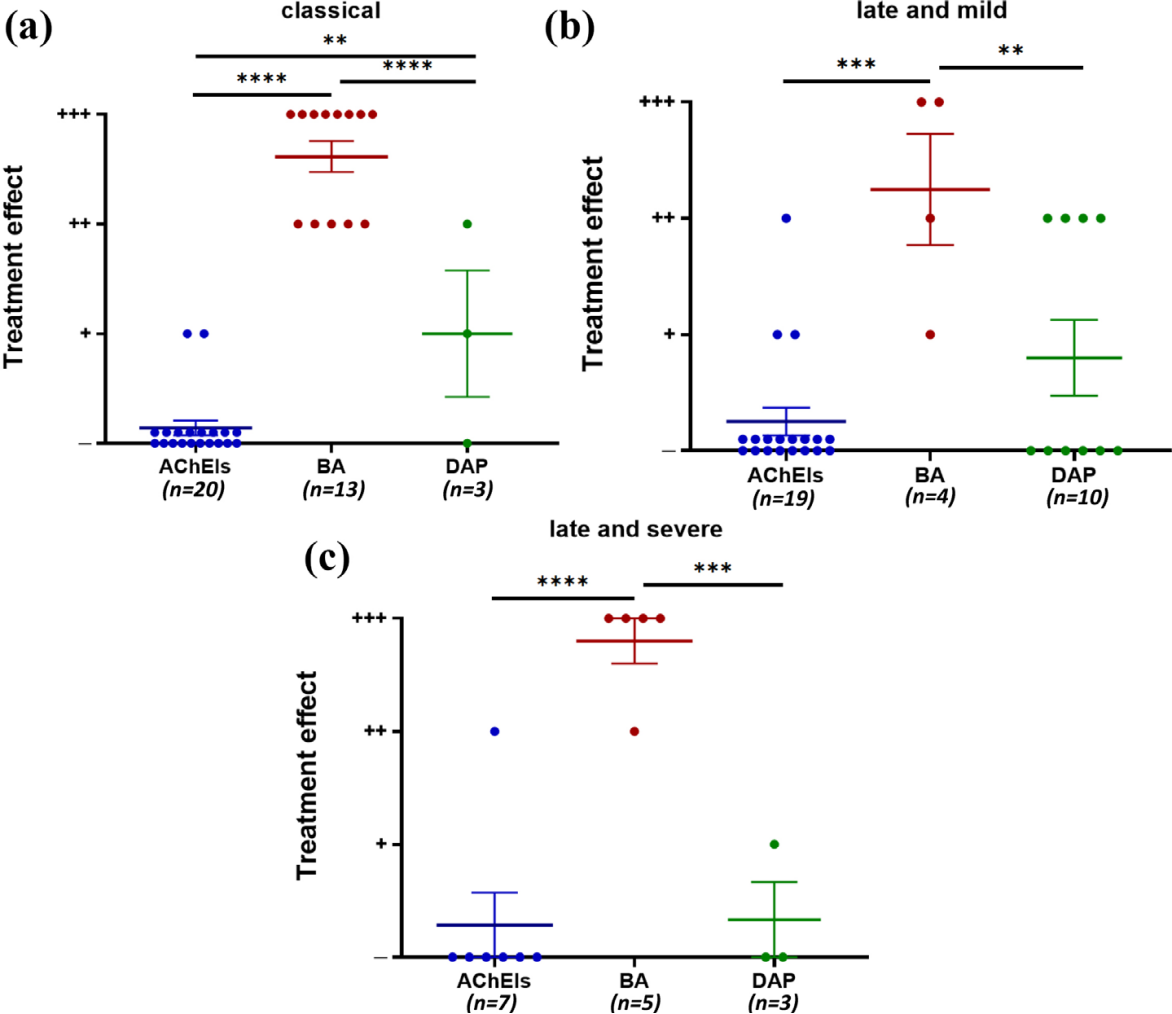
Treatment effects in CMS patients with *COLQ* mutations by phenotype. Treatment effects were categorized as noted in Fig. 4. DAP, 3,4-diaminopyridine; BA, β2-adrenergic receptor agonist; AChEI, acetylcholinesterase inhibitor; FLX, fluoxetine; GC, glucocorticoid. The phenotype of “early and mild” is not displayed due to its limited number of patients (*n* = 7). Treatment effects for (**a**) the “classical” phenotype, (**b**) the “late and mild” phenotype, and (**c**) the “late and severe” phenotype. Each point represents one patient. Mean and SEM are indicated. **p < 0.01, ***p < 0.001 and ****p < 0.0001 by the Kruskal-Wallis test.

β-adrenergic receptor agonists showed a better effect than AChEI and DAP for treating patients with the “classical” phenotype. DAP showed a better effect than AChEIs (Fig. **6a**). For treating patients with the “late and mild” phenotype, β-adrenergic receptor agonists showed better effects than AChEIs and DAP, and FLX showed better effects than AChEIs and DAP (Fig. **6b**). For treating patients with the “late and severe” phenotype, only β-adrenergic receptor agonists, AChEIs, and DAP were used, and β-adrenergic receptor agonists showed the best effect (Fig. **6c**). Some patients underwent more than one kind of treatment. No significant difference was found in the treatment of patients with “early and mild” phenotypes owing to the limited data size.

### 3.5 Influence of Sex on Treatment Effect

**Fig. (7):**
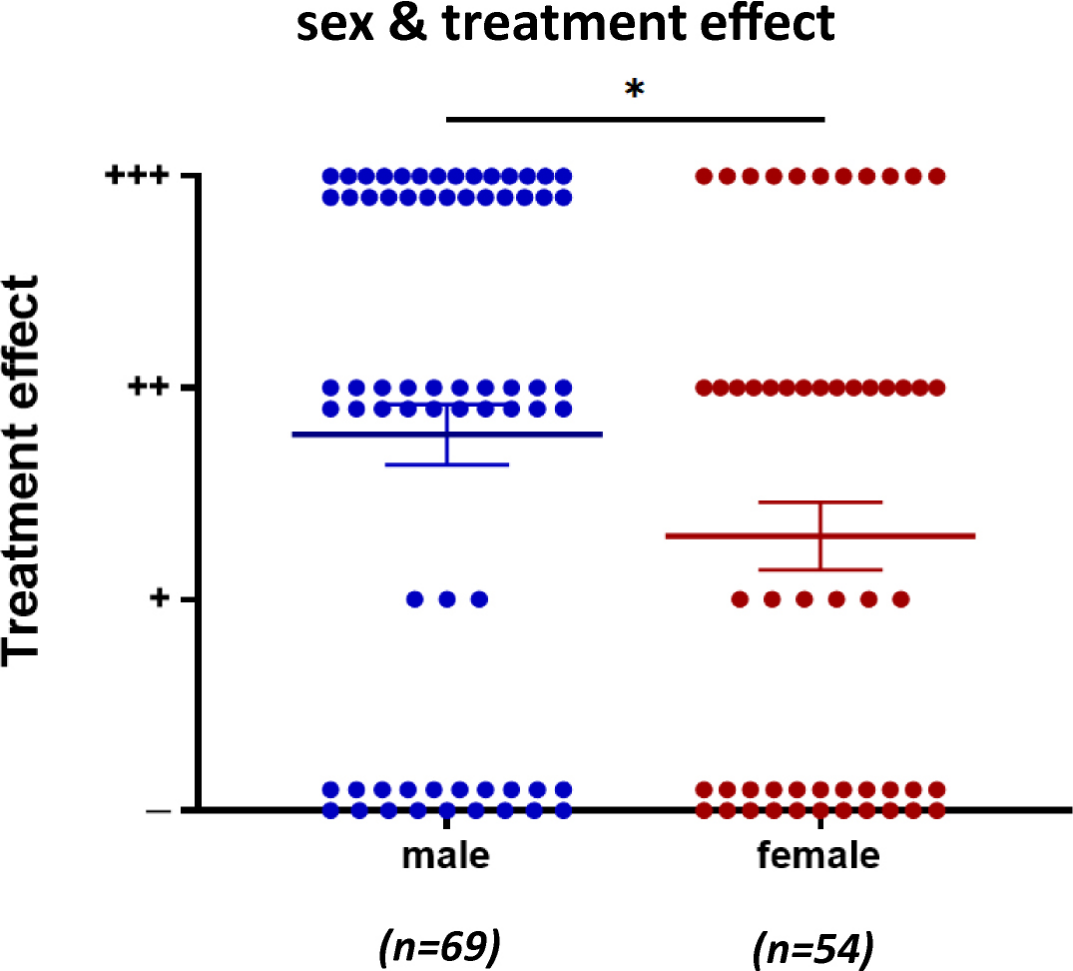
Treatment effects in CMS patients with *COLQ* mutations by sex. Treatment effects were categorized as noted in Fig. 4 Each point represents one patient. Mean and SEM are indicated. *p < 0.05 by the Mann-Whitney test.

Among the enrolled patients, 123 were referred for sex and clear treatment effects (69 males and 54 females). Male patients showed better treatment effects than female patients (Fig. **7**).

### 3.6 Influence of Age at Symptom Onset on Treatment Effect

**Fig. (8):**
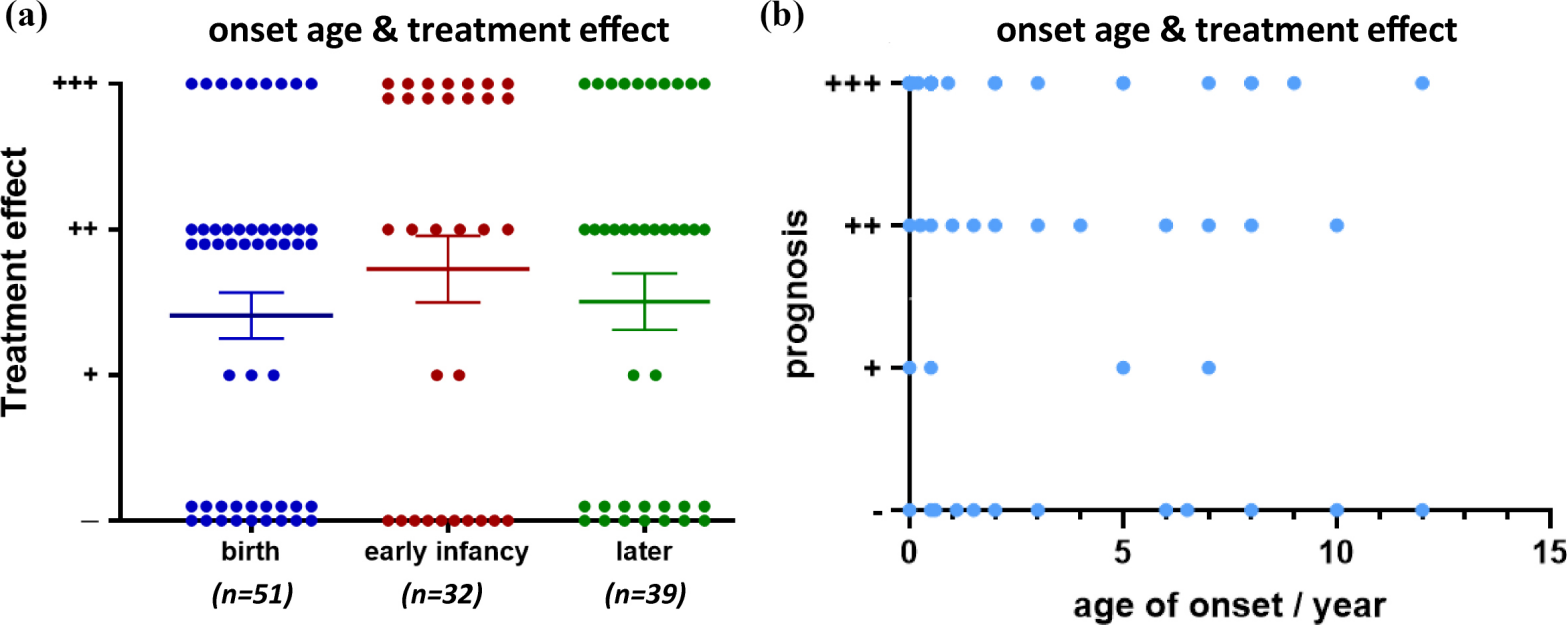
Treatment effect in CMS patients with *COLQ* mutations by onset age. Treatment effects were categorized as noted in Fig. **4**. Mean and SEM are indicated. (**a**) Patients were divided into three groups according to onset age. (*p* > 0.05 by the Kruskal-Wallis test). (**b**) Simple linear regression was used to analyze the relation between onset age and treatment effect. (*p* > 0.05 by the Kruskal-Wallis test).

Among all enrolled patients, clear information regarding onset age and treatment effect was provided for 122 patients. Firstly, we classified the patients into three groups according to their age when the symptoms onset: the “birth” group (onset at birth, *n* = 51), the “early infancy” group (onset after birth and before six months, *n* = 32), and the “later” group (onset after six months, *n* = 39). Then, simple linear regression was used for analysis. Onset age was not found to have an influence on treatment effect in this study (Fig. **8a** and **b**).

## 4 DISCUSSION

A deficiency of endplate AChE caused by *COLQ* mutations, which impedes ACh elimination in the NMJ, is one of the major causes of CMS. We reviewed the mutation types and treatment information of patients identified to date. We found that β-adrenergic receptor agonists performed best among the pharmacological methods used. AChEIs, such as pyridostigmine and neostigmine, hardly benefit patients, and in many cases, may even worsen their treatment outcomes. 3,4-DAP, GCs, and FLX perform better than AChEIs but not β-adrenergic receptor agonists. Patients with CMS with *COLQ* mutations were divided into four phenotypes according to the severity of symptoms and onset age [12, 22, 27-30]. However, no significant difference in treatment effect was found in these phenotypes. β-adrenergic receptor agonist administration was found to be the optimal treatment choice for all patients. Given the available data, male patients were found to respond better to treatment than female patients. No significant correlation was found between onset age and treatment effect. In addition, no significant difference in treatment effect was found between patients with different *COLQ* mutation types (Supplementary Fig. **S1**).

NMJs are highly specialized chemical synapses between motor neurons and muscle fibers that transform electrical impulses into ACh release. ACh binds to AChR at the endplate, causing the opening of sodium channels and muscle contraction [62]. Typically, AChE breaks down ACh and effectively terminates synaptic transmission. However, as shown in Fig. **1**, *COLQ* mutations can impair trimeric organization (MuSK-AChE-COLQ trimeric organization), truncate collagen domains, or influence the interaction between COLQ and MuSK. These factors can cause endplate AChE deficiency, which prolongs the half-life of ACh and leads to NMT blockade due to receptor desensitization [3].

AChEIs, including pyridostigmine and neostigmine, inhibit ACh from breaking down to choline and acetate, thus prolonging the level and duration of action of the neurotransmitter ACh. As a frequently used drug for treating CMS, AChEIs benefit CMS patients with pre-synaptic mutations, such as *SLC5A7, CHAT, SLC18A3, VAMP1, SYB1, SYT2,* and *MUNC13-1*; synaptic mutations, such as *LAMA5*; and postsynaptic mutations, such as *CHRNA1, FCCMS, MYO9A, PREPL, PLEC1*, and *CHRND*. In addition, AChEIs can help some patients with defects in glycosylation, which is a common post-translational modification that participates in the formation, stabilization, and function of the NMJ [63, 64]. Glycosylation defects can be caused by mutations in *GFPT1*, *GMPPB*, *ALG2*, *ALG14*, and *DPAGT1*, which hampers the solubility, folding, stability, assembly, and intracellular transport of peptides and induces NMT defects [3, 65, 66]. However, in patients with CMS caused by *COLQ* mutations, AChEIs increase excess ACh. As a result, AChEIs do not have a significant positive effect and may have negative effects. Therefore, these drugs should be avoided once patients with CMS have been verified to have *COLQ* mutations.

3,4-DAP blocks the presynaptic voltage-gated potassium channel complex, which prolongs nerve terminal depolarization and promotes ACh release from the nerve terminal into the synaptic cleft [3, 67]. It has been proven to be effective in treating patients with CMS with *SNAP25, SYT2, MUNC13-1, LAMA5, COL13A1, CHRND, RAPSN, SLC25A1,* or *DPAGT1* mutations [66]. Interestingly, as shown in Fig. (**4**), some patients with *COLQ* mutations benefit from 3,4-DAP, which is contrary to the opinion that 3,4-DAP may worsen CMS in patients with *COLQ* mutations. However, the mechanism remains unclear. One possible explanation reported in previous research is an increase in muscle force caused by 3,4-DAP blocking delayed rectifier potassium channels, which prolongs the action potential by slowing repolarization and increasing intracellular Ca^2+^ levels [68]. Increased Ca^2+^ directly enhances muscular strength without affecting the NMJ. Further studies are required to determine the underlying mechanisms.

FLX is a selective serotonin reuptake inhibitor used to treat depression [69]. It is also a long-lived AChR open-channel blocker that prevents depolarization and AChR desensitization by decreasing prolonged synaptic currents [3]. This may explain why FLX performed significantly better than the AChEIs.

In our study, β-adrenergic receptor agonists performed best among all pharmacological methods for treating patients with CMS due to *COLQ* mutations. These drugs are widely used in clinical practice. The beneficial effect of ephedrine in patients with myasthenia gravis has been recognized since the 1930s [70]. Physicians reported its effect on improving muscle strength and decreasing fatigability when treating dysmenorrhea by chance [71, 72], but anticholinesterases and corticosteroids have since replaced it owing to possible adverse effects [73]. Ephedrine returned to the spotlight again owing to its effect on CMS treatment, especially CMS caused by *DOK7* mutations. DOK7 is a muscle-intrinsic activator of MuSK. Mutations in *DOK7* may lead to DOK7 deficiency, thus leading to NMJ defects [3, 74, 75]. Although β-adrenergic receptor agonists have been proven effective in different subtypes of CMS, the exact mechanism remains unclear. β-adrenergic receptor agonists have been proven to be effective in most CMS patients with mutations such as *SLC5A7*, *LAMB2*, *COL13A1*, *CHRNE*, *DOK7*, and *MUSK* [32, 66, 76], but they have no effect in CMS patients with *SYT2* [77], *TOR1AIP1* [78], and *CHD8* [79] mutations.

Researchers have proved that salbutamol directly affects proteins located at the endplate and increases the number of AChR clusters in the C2C12 cell model of *DOK7*, thus stabilizing AChR clusters [80]. In a zebrafish model of Dok7-deficient or MuSK-deficient CMS, salbutamol reduced motility defects and improved AChR clustering and the size of synaptic contacts. These effects can be prevented using a selective β2 antagonist [81]. The mouse model of CMS due to the *Colq* mutation showed gradual improvement in muscle strength and several postsynaptic morphological defects after salbutamol treatment, suggesting that β-adrenergic receptor agonists affect the endplate [82]. One possible reason for this effect may be that β-adrenergic receptor agonists activate the cyclic AMP-protein kinase A cascade in muscle fibers, which participates in synaptic integrity and AChR stability [83]. Moreover, β-adrenergic receptor agonists have been proven to have a therapeutic effect on skeletal muscle abnormalities, including increasing skeletal muscle while decreasing body fat, attenuating muscular waste, and enhancing muscular repair and growth [84]. Chronic β-adrenergic receptor agonist administration has been shown to promote skeletal muscular hypertrophy, especially in type II fibers [85, 86].

In evidence-based medicine, systematic reviews of multiple RCTs provide the most reliable evidence to inform clinical practice. However, for disorders such as CMS with a notably low incidence rate and high subtype variation, carrying out RCTs is almost impossible, and there are no RCT on CMS thus far. Therefore, a meta-analysis of clinical knowledge regarding the efficacy of pharmacological treatments and other types of interventions based on observational studies and case reports is necessary. However, there were some limitations to our meta-analysis. (1) There might have been publication bias due to the possibility that only positive results were published. (2) Most reports that we reviewed lacked a quantitative assessment of treatment effectiveness. Instead, they were described qualitatively using words such as negative, partial, and positive. This may also lead to bias and flatten variations in reported treatment outcomes. (3) Insufficient information was provided regarding drug doses, so it was difficult to discern dose-effect relationships for the investigated treatments.

## CONCLUSION

Our meta-analysis provided evidence that β-adrenergic receptor agonists, including salbutamol and ephedrine, can be used as first-line pharmacological treatments for CMS patients with *COLQ* mutations. We recommend that once a CMS patient is identified with a *COLQ* mutation, the clinician should stop administering AChEIs and provide β-adrenergic receptor agonists, such as salbutamol and ephedrine, for improved prognosis.

## Figures and Tables

**Fig. (2):**
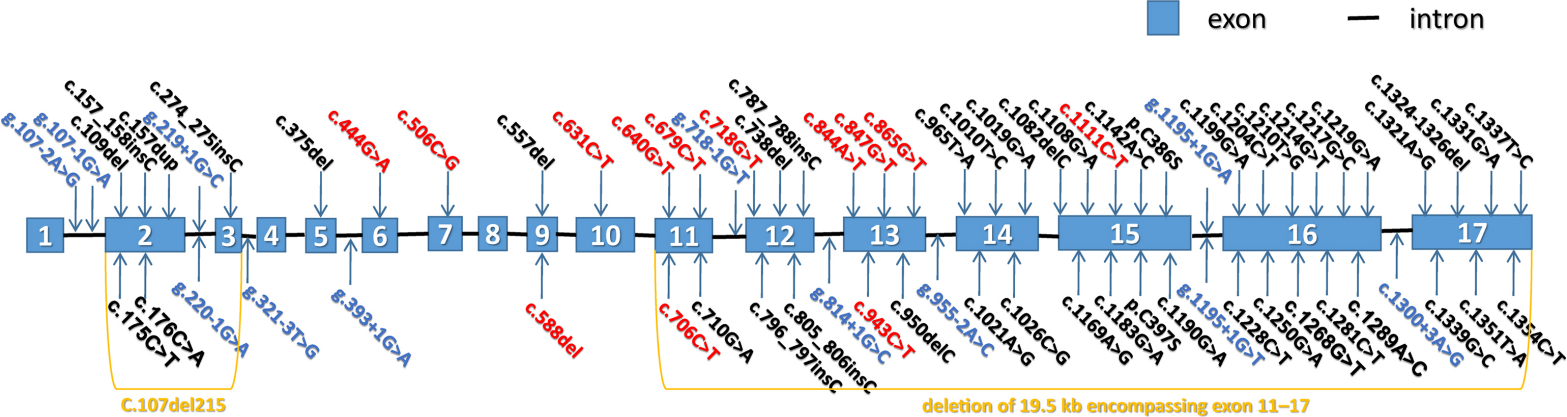
Mutations of *COLQ* identified so far. The blue boxes represent exons, the black lines represent introns; exons and introns are not drawn to scale. The splice-site mutations are marked in blue, the truncating mutations are marked in red, missense mutations are marked in black, and two multi-exon deletions are also shown in khaki. These mutations are described referring to NM_005677.4,.(*A is available in the electronic copy of the article*).

**Fig. (3):**
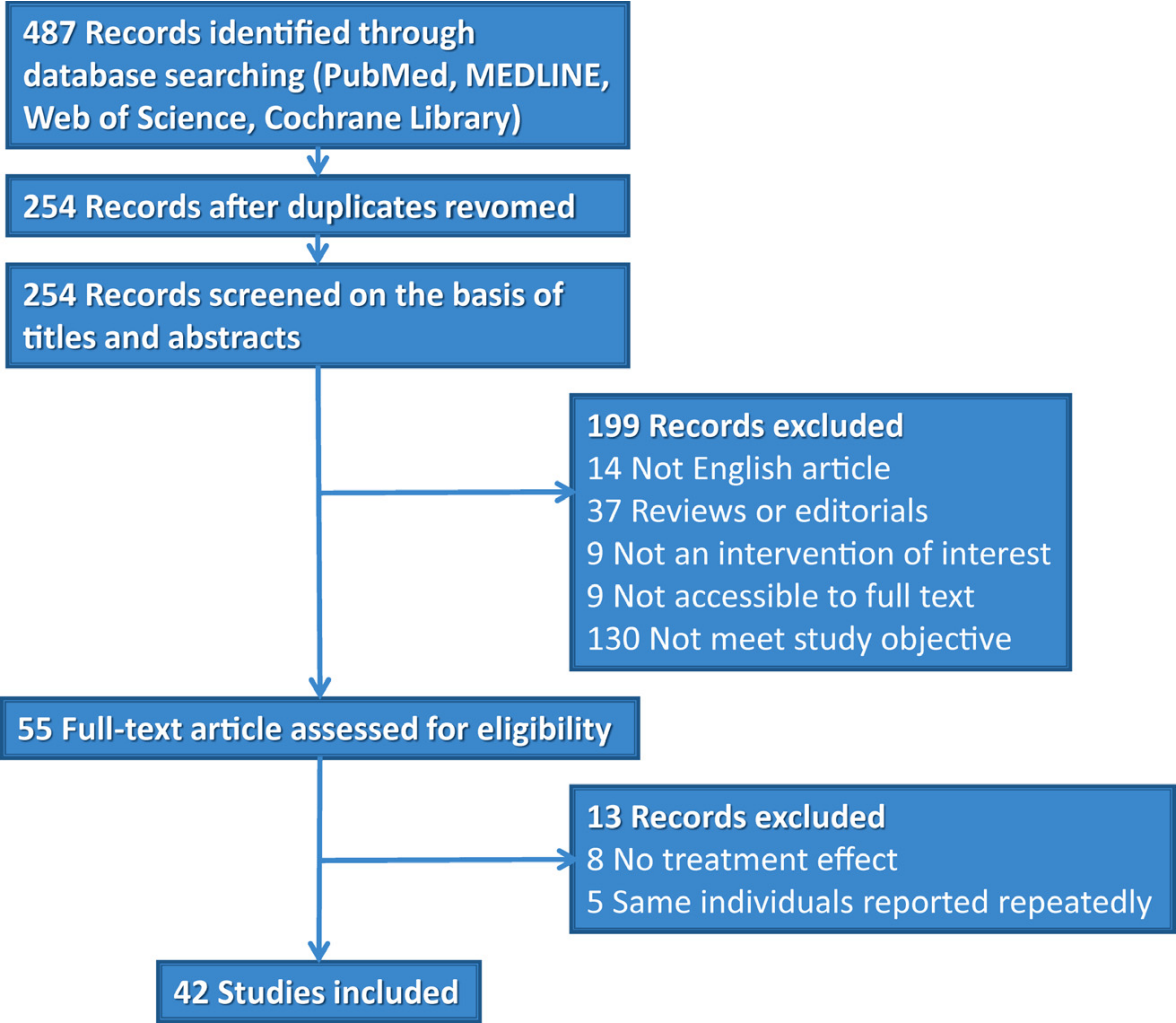
Flow chart illustrating the study selection process for systematic review and meta-analysis.

**Table 1:** Different pharmacological strategies used in CMS patients with *COLQ* mutations.

**Category**	**Drug**	**Number of Use**
AChEIs	Pyridostigmine, neostigmine, pyridostigmine bromide, edrophonium	*n* = 116
BA	Salbutamol, levosalbutamol, ephedrine, terbutaline	*n* = 75
DAP	3,4-diaminopyridine	*n* = 23
FLX	Fluoxetine	*n* = 6
GCs	Methylprednisolone, prednisolone, prednisone	*n* = 5
Others	L-Carnitine + Coenzyme Q10 [17]; cocktail of carnitine, thiamine, riboflavin, menadione and Coenzyme Q [86]; levetiracetam, carbamazepine, midazolam, clonazepam, vitamin B6, and vigabatrin [13]; guanidine [27]	*n* = 7

**Note:** Some patients underwent more than one kind of treatment.

**Abbreviations:** AChEI, acetylcholinesterase inhibitor; BA, β2-adrenergic receptor agonist; DAP, 3,4-diaminopyridine; FLX, fluoxetine; GC, glucocorticoid.

## LIST OF ABBREVIATIONS

AChE: Acetylcholinesterase
AChR: Acetylcholine Receptor
CMS: Congenital Myasthenic Syndromes
FLX: Fluoxetine
GCs: Glucocorticosteroids
NMJ: Neuromuscular Junction
RCTs: Randomized Clinical Trials
VGKCs: Voltage-gated Potassium Channels

## SUPPLEMENTARY MATERIAL

PRISMA checklist is available as supplementary material on the publisher’s website along with the published article.
