# Supplementary material for: Pharmacological Treatments for Congenital Myasthenic Syndromes Caused by COLQ Mutations
Source: Curr Neuropharmacol. 2023 May 18;21(7):1594–605. doi: 10.2174/1570159X21666230126145652 (PMC10472815; doi:10.2174/1570159X21666230126145652)
Supplement: Supplementary file 1 [file CN-21-1594_SD1.pdf]

Supplementary Material

Pharmacological Treatments for Congenital Myasthenic Syndromes Caused by *COLQ* Mutations

Shuai Shao[1,2,3], Guanzhong Shi[1,2,3], Fang-Fang Bi[1,3] and Kun Huang[1,3,*]

[1]*Department of Neurology, Xiangya Hospital, Central South University, Changsha, Hunan Province, China;* [2]*Xiangya School of Medicine, Central South University, Changsha, Hunan province, China;* [3]*National Clinical Research Center for Geriatric Disorders, Xiangya Hospital, Central South University, Changsha, Hunan Province, China*

1875-6190/23 $65.00+.00 **© 2023 Bentham Science Publishers**

Supplementary Table 1. Information of identified mutations of *COLQ* so far

Mutation	Location	Consequence	References
g.107-2A>G	intron 1	not mentioned	[1]
g.107-1G>A	intron 1	affecting the exon encoding PRAD, associated with 788insC and R236X and may impair trimeric organization	[2, 3]
c.109del	exon 2	not mentioned	[3]
c.157dup	exon 2	frame shift mutation (p.Leu53ProfsX81)	[4]
c.157_158insC	exon 2	not mentioned	[3]
c.175C>T	exon 2	missense substitution	[4]
c.176C>A	exon 2	changing the fourth proline in a stretch of five prolines in PRAD	[5]
c.107_215del	exon 2,3	skipping of exons 2 and 3, causing a frameshift after codon 35, abolishing PRAD and the following domains	[6]
g.219+1G>C	intron 2	splice mutation	[4]
g.220-1G>A	intron 2	disrupting the invariant last base of the acceptor splice site at the start of exon 3, skipping the in-frame exon 3 or creating a new acceptor splice site 13 bp downstream, resulting in a truncated or absent protein	[7, 8]
c.274_275insC	exon 3	20 missense codons followed by a stop codon	[9]
g.321-3T>G	intron 3	causing exon skipping and non-functional gene products	[10]
c.375del	exon 5	frameshift after codon 125 in collagen domain, stop codon after 37 missense codons	[5]
g.393+1G>A	intron 5	causing exon skipping and non-functional gene products	[10, 11]
c.444G>A	exon 6	truncating the collagen domain of COLQ	[12]
c.506C>G	exon 7	loss of the distal two-thirds of the collagen domain	[6]
c.557del	exon 9	not mentioned	[13]
c.588del	exon 9	causing absent or disrupted protein products	[7]
c.631C>T	exon 10	nonsense mutation in the ColQ collagen domain	[9]
c.640G>T	exon 11	truncating ColQ in the distal third of the collagen domain	[6, 14]
c.679C>T	exon 11	nonsense mutation	[4, 15]
c.706C>T	exon 11	nonsense mutation, producing truncated chains which cannot trimerize	[2-4]
c.710G>A	exon 11	substitution of a highly conserved residue in one of the heparin sulphate proteoglycan-binding domains	[3]
chromosome deletion*	exon 11–17	deletion of 19.5 kb encompassing exon 11–17; truncating half of the collagen domain	[16]
g.718-1G>T	intron 11	not mentioned	[11]
c.718G>T	exon 12	truncating ColQ before C-terminal region and preventing the formation of asymmetric AChE	[9]
c.738del	exon 12	not mentioned	[3]
c.787_788insC	exon 12	frameshift after codon 262 predicting 36 missense codons followed by a stop codon, sparing 85% of the collagen domain	[4, 6, 14]
c.796_797insC	exon 12	not mentioned	[3]
c.805_806insC	exon 12	frameshift after codon 269 in collagen domain, stopping codon after 31 missense codons	[5]
g.814+1G>C	intron 12	affecting the invariant cytosine and adenine donor splice-site of intron 12	[17]
c.844A>T	exon 13	truncating ColQ at 10 codons upstream to the C-terminal end of the collagen domain	[6]
c.847G>T	exon 13	loss of the downstream C terminal domain and loss of function	[18]
c.865G>T	exon 13	truncating ColQ and preventing formation of asymmetric AChE	[11]
c.943C>T	exon 13	eliminating 86% of the residues of the C-terminal region	[19]
c.950del	exon 13	frameshift in exon 13 and truncating the collagen-encoding region	[3, 20]
g.955-2A>C	intron 13	not mentioned	[21]
c.965T>A	exon 14	compromising the tertiary structure of C-terminal domain and defective triple helix formation	[22]

c.1010T>C	exon 14	impairing trimeric organization and its anchoring within the synaptic basal lamina, interfering with the binding of ColQ interacting proteins	[23]
c.1019G>A	exon 14	missense substitution	[4]
c.1021A>G	exon 14	not mentioned	[3]
c.1026C>G	exon 14	producing some asymmetric AChE species	[5]
c.1082del	exon 15	frameshift after codon 360, 64 missense codons followed by a stop codon, abolishing the C-terminal domain of ColQ	[6]
c.1108G>A	exon 15	disrupting the interaction of ColQ with MuSK, and with basement membrane extract	[24]
c.1111C>T	exon 15	producing some asymmetric AChE species	[5]
c.1142A>C	exon 15	impairing anchoring of the enzyme within the synaptic basal lamina	[25]
p.C386S(1156T> A;1157G>C) **	exon 15	disturbing the anchoring of ColQ to the basal lamina	[3]
c.1169A>G	exon 15	likely pathogenic	[26]
c.1183G>A	exon 15	substitution of a highly conserved aspartic acid for asparagine	[27]
p.C397S(1189T> A;1190G>C) **	exon 15	disrupting the interaction of ColQ with MuSK, and with basement membrane extract	[24]
c.1190G>A	exon 15	missense mutation	[28, 29]
g.1195+1G>A	intron 15	skipping of exon 15, frameshift after codon 358 in C-terminal region, stop codon after 26 missense codons; insertion incompetence, impaired triple helix formation	[5, 14, 30]
g.1195+1G>T	intron 15	affect splicing, maybe similar to IVS15+1G>A	[3]
c.1199G>A	exon 16	disrupting the interaction of ColQ with MuSK, and with basement membrane extract	[24]
c.1204C>T	exon 16	missense variant in the C-terminal domain	[18]
c.1210T>G	exon 16	disrupting the interaction of ColQ with MuSK, and with basement membrane extract	[24]
c.1214G>T	exon 16	disrupting the interaction of ColQ with MuSK, and with basement membrane extract	[24]
c.1217G>C	exon 16	affecting a conserved amino acid and is being described in conjunction with a pathogenic variant	[7]
c.1219G>A**	exon 16	producing some asymmetric AChE species	[5]
c.1228C>T***	exon 16	missense mutation	[4]
c.1250G>A	exon 16	disturbing the anchoring of ColQ to the basal lamina	[3]
c.1268G>T	exon 16	damaging the tertiary structure, creating two new H-bonds, disrupting the interaction of ColQ with MuSK, and with basement membrane extract	[24]
c.1281C>T	exon 16	predicted to cause p.Cys427Cys, also creating a cryptic splice donor site in exon 16 leading to an aberrant splicing and the deletion of 19 nucleotides in exon 16 confirmed by mRNA analysis	[4]
c.1289A>C	exon 16	heralding a later onset and slow progression	[3, 4, 30, 31]
c.1300+3A>G	intron 16	skipping exon 16, eliminating 34% of the native residues of the C-terminal region and resulting in 57 missense codons followed by a stop codon	[19]
c.1321A>G	exon 17	a complete absence of asymmetric, collagen-tailed AChE forms in patients	[32]
c.1324-1326del	exon 17	not mentioned	[3]
c.1331G>A	exon 17	producing some asymmetric AChE species	[5]
c.1337T>C	exon 17	missense substitution	[4]
c.1339G>C	exon 17	decreasing the activities of ColQ-tailed AChE bound to MuSK to 50% or less	[22]
c.1351T>A	exon 17	missense substitution	[4]
c.1354C>T	exon 17	decreasing the activities of ColQ-tailed AChE bound to MuSK to 50% or less	[22]

*The chromosome deletion mentioned above resulting in the deletion of 19.5 kb encompassing exon 11–17 is called arr[GrCh37] 3p25.1 (15491478x1, 15492150_15511615x0, 15511740x1) [16].

**The amino acid mutations that can be caused by different cDNA mutations, without enough information in the original papers for us to identify the specific one [3, 24].

***There might be some conflict in the sequence of amino acids or bases between R410Q(1219G>A) [5] and R410W(1228C>T) [4]. According to the cDNA sequence of the human *COLQ* gene by Kinji Ohno [6], the latter number of amino acids should be correct.

This table showed identified mutations of *COLQ* so far, following the nomenclature guidelines by the Human Genome Variation Society (HGVS) of the Human Genome Organization (HUGO) at https://varnomen.hgvs.org[33], and referring to NM_005677.4.

Supplementary Table 2. Qualitative assessment of the included studies

	**Domains for Evaluating the Methodological Quality of Case Reports and Case Series**							
	**Selection**	**Ascertainment**		**Causality**				**Reporting**
**References**	**Q1**	**Q2**	**Q3**	**Q4**	**Q5**	**Q6**	**Q7**	**Q8**
Mohammad et al [34]	Y	Y	Y	Y	N	N	N	Y
Violeta et al [3]	Y	Y	Y	Y	N	N	Y	Y
Rashid et al [27]	Y	Y	Y	Y	N	N	Y	Y
Hussein et al [23]	Y	Y	Y	Y	N	N	Y	N
Guven et al [35]	Y	Y	Y	Y	N	N	Y	Y
Wargon et al [4]	Y	Y	Y	Y	Y	N	Y	Y
Hansashree et al [17]	Y	Y	Y	Y	N	N	Y	Y
Chee et al [7]	Y	Y	Y	Y	N	N	Y	Y
Laforgia et al [16]	Y	Y	Y	Y	N	N	Y	Y
Pallithanam et al [13]	Y	Y	Y	Y	N	N	N	N
Vidanagamage et al [36]	Y	Y	Y	Y	N	Y	Y	Y
Shapira et al [9]	Y	Y	Y	Y	N	N	N	N
Nalinee et al [11]	Y	Y	Y	Y	N	N	Y	Y
Duran et al [37]	Y	Y	Y	Y	N	N	N	N
Vaibhav et al [38]	Y	Y	Y	Y	N	N	Y	N
Bestue-Cardiel et al [30]	Y	Y	Y	Y	N	Y	Y	N
Wai-Lan et al [10]	Y	Y	Y	Y	N	N	Y	Y
Ohno et al [5]	Y	Y	Y	Y	N	N	N	N
Müller et al [32]	Y	Y	Y	Y	N	N	N	Y
Ohno et al [19]	Y	Y	Y	Y	N	N	N	N
Pavalan et al [18]	Y	Y	Y	Y	N	N	N	N
Devin et al [15]	Y	Y	Y	Y	N	N	N	Y
Caggiano et al [39]	Y	Y	Y	Y	N	N	N	N
Benito et al [14]	Y	Y	Y	Y	N	N	N	Y
Gülen et al [26]	Y	Y	Y	Y	N	N	Y	N
Yawen et al [40]	Y	Y	Y	Y	N	N	N	N
Yiran et al [41]	Y	Y	Y	Y	N	N	N	Y
Sophelia et al [8]	Y	Y	Y	Y	Y	Y	Y	Y
Justin et al [42]	Y	Y	Y	Y	N	N	N	N
Hacer Durmus et al [12]	Y	Y	Y	Y	N	N	N	N
Uluç Yiş et al [43]	Y	Y	Y	Y	N	N	N	N
Bestue-Cardiel et al [30]	Y	Y	Y	Y	N	N	N	N
Qingyun et al [28]	Y	Y	Y	Y	N	N	N	Y
Ohon et al [6]	Y	Y	Y	Y	N	N	N	Y
Donger et al [31]	Y	Y	Y	Y	N	N	N	N
Tomohiko et al [22]	Y	Y	Y	Y	N	N	N	N
Arredondo et al [24]	Y	Y	Y	Y	N	N	N	N
Mishra et al [21]	Y	Y	Y	Y	N	N	N	N

Albassam et al [44]	Y	Y	Y	Y	N	N	N	N
Anna et al [1]	Y	Y	Y	Y	N	N	Y	N
Joshi et al [45]	Y	Y	Y	Y	N	N	N	N
Hutchinson et al [46]	Y	Y	Y	Y	N	Y	N	Y
Eduardo et al [47]	Y	Y	Y	Y	N	N	N	N

Selection: 1. Does the patient(s) represent(s) the whole experience of the investigator (center) or is the selection method unclear to the extent that other patients with similar presentation may not have been reported?
Ascertainment: 2. Was the exposure adequately ascertained? 3. Was the outcome adequately ascertained?
Causality: 4. Were other alternative causes that may explain the observation ruled out? 5. Was there a challenge/rechallenge phenomenon? 6. Was there a dose–response effect? 7. Was follow-up long enough for outcomes to occur?
Reporting: 8. Is the case(s) described with sufficient details to allow other investigators to replicate the research or to allow practitioners make inferences related to their own practice?

Supplementary Table 3. Information of 164 enrolled patients.

No	Ref	GS	Mutation	Position	Gender	Onset Age	IM/NPD/I/MD/V	Treatment	Prognosis
1	[34]	HIR	NA	NA	F	EI	Y/Y/Y/Y/0	BA+++	+++
2	[3]	HIR	c.1250G>A	E16	F	Birth	0/N/Y/Y/N	AChEIs-/BA++	++
3		HIR	c.631C>T;g.1195+1G>T	E10;I15	F	Birth	0/N/0/Y/N	AChEIs-	-
4		HIR	c.1082del	E15	M	Birth	0/N/0/Y/Y	AChEIs-/BA++	++
5		HIR	c.1289A>C	E16	F	6	0/0/0/N/Y	AChEIs-/BA++/DAP-	++
6		HIR	c.1082del	E15	F	EI	0/0/0/Y/0	AChEIs+	+
7		HIR	c.1289A>C	E16	F	6	0/0/0/N/0	AChEIs-/DAP++	++
8		HIR	g.107-1G>A;c.950del	I1;E13	F	Birth	0/0/0/Y/0	AChEIs-	-
9		HIR	g.107-1G>A;c.950del	I1;E13	M	Birth	0/0/Y/Y/Y	NA	NA
10		HIR	c.1321A>G	E17	F	EI	0/0/0/Y/0	AChEIs-/DAP-	-
11		HIR	c.1289A>C	E16	M	EI	0/0/0/N/0	AChEIs-/DAP-	-
12		HIR	c.1289A>C	E16	F	7	0/0/0/N/0	AChEIs-/DAP-	-
13		HIR	c.1021A>G	E14	F	EI	0/0/Y/N/0	AChEIs++/DAP-	++
14		HIR	c.157_158insC	E2	M	Birth	0/0/0/Y/0	AChEIs-	-
15		HIR	c.109del;c.1324-1326del	E2;E17	M	2	0/0/0/Y/0	AChEIs-	-
16		HIR	c.679C>T	E11	F	EI	0/0/0/Y/0	AChEIs+	NA
17		HIR	c.444G>A;p.C386S	E6;E15	M	Birth	0/N/0/Y/Y	AChEIs+	+
18		HIR	c.706C>T	E11	F	Birth	0/0/0/Y/Y	AChEIs+/BA+++	+++
19		HIR	c.1082del	E15	F	Birth	0/0/0/Y/0	AChEIs-	-
20		HIR	c.710G>A;c.796_797insC	E11;E12	M	Birth	0/0/0/Y/0	AChEIs-	-
21		HIR	c.738del	E12	M	Birth	0/0/0/Y/Y	BA++	++
22		HIR	c.679C>T	E11	F	EI	0/0/0/Y/0	NA	NA
23		HIR	g.219+1G>C	I2	M	EI	0/0/0/Y/N	AChEIs++	++
24	[27]	HIR	c.1183G>A	E15	M	Birth	0/Y/0/0/Y	AChEIs-/BA+++/DAP+	+++
25	[23]	HIR	c.1010T>C	E14	F	Birth	Y/Y/N/Y/Y	AChEIs-	-
26		HIR	c.1010T>C	E14	F	EI	Y/0/Y/0/Y	AChEIs-	-
27	[35]	LMR	c.444G>A	E6	M	EI	Y/0/N/0/0	BA+++/DAP++	+++
28		LMR	c.444G>A	E6	F	EI	N/0/Y/0/0	BA++	++
29		LMR	c.444G>A	E6	F	Birth	N/0/N/0/0	AChEIs-/BA++/DAP++	++
30		LMR	c.444G>A	E6	M	Birth	Y/0/N/0/0	AChEIs-/BA-/DAP++	++
31	[4]	HIR	g.107-1G>A;c.706C>T	I2;E11	F	EI	0/0/0/0/Y	AChEIs-/BA+++/DAP-/OA-	+++
32		HIR	c.1281C>T	E16	M	10	Y/0/0/0/N	AChEIs-/DAP++	++
33		HIR	c.1289A>C	E16	F	Birth	0/0/0/0/N	AChEIs-/DAP-/OA++	++
34		HIR	g.107-1G>A;c.787_788insC	I2;E12	F	Birth	0/0/0/0/N	AChEIs-/OA++	++
35		HIR	c.1281C>T;c.1289A>C	E16	F	3	Y/0/0/0/N	DAP++	++
36		HIR	c.1082del;c.1337T>C	E15;E17	M	EI	0/0/0/0/N	AChEIs-	-
37		HIR	c.157dup	E2	F	Birth	0/0/0/0/Y	AChEIs-/DAP-/BA+++	+++

38		HIR	c.175C>T;c.1351T>A	E2;E17	M	2	0/0/0/0/Y	AChEIs-/FLX++	++
39		HIR	c.679C>T	E11	M	2	Y/0/0/0/N	AChEIs-/DAP-/BA+++	+++
40		HIR	c.444G>A	E6	M	Birth	Y/0/0/0/Y	AChEIs-/DAP-	-
41		HIR	c.1289A>C	E16	M	6	Y/0/0/0/N	AChEIs-	-
42		HIR	c.1228C>T	E16	F	2	Y/0/0/0/N	NA	NA
43		HIR	g.219+1G>C;c.1019G>A	I2;E14	F	2	0/0/0/0/Y	AChEIs-/OA+	+
44		HIR	g.219+1G>C;c.1019G>A	I2;E14	M	13month	0/0/0/0/N	NA	NA
45		HIR	c.1082del;c.1321A>G	E15;E17	M	Birth	0/0/0/0/N	AChEIs-/DAP++	++
46	[17]	LMR	NA	NA	M	EI	Y/Y/N/Y/0	AChEIs++	++
47	[7]	LMR	g.220-1G>A；c.1217G>C	I2;E16	M	Birth	N/Y/Y/0/Y	AChEIs-/GCs-/BA+++	+++
48		LMR	c.588del	E9	F	Birth	Y/Y/N/0/Y	AChEIs-/BA+	+
49	[16]	HIR	NA	E11–17	M	EI	Y/0/0/Y/Y	AChE-Is+/BA++/DAP++/OC+	++
50	[13]	LMR	c.557del	E9	M	Birth	N/Y/0/0/Y	AChEIs-/BA++	++
51	[36]	LMR	c.1228C>T	E16	M	8	N/N/N/N/Y	FLX+++/BA+	+++
52	[9]	HIR	c.274_275insC；c.631C>T	E3;E10	F	Birth	N/0/0/0/0	NA	NA
53		HIR	c.718G>T	E12	M	Birth	Y/0/0/0/0	AChEIs-	-
54		HIR	c.718G>T	E12	M	EI	N/0/0/0/0	AChEIs-	-
55		HIR	c.718G>T	E12	F	EI	N/0/0/0/0	AChEIs-	-
56		HIR	c.718G>T	E12	M	EI	N/0/0/0/0	AChEIs-	-
57		HIR	c.718G>T	E12	M	EI	N/0/0/0/0	AChEIs-	-
58		HIR	c.718G>T	E12	M	2	N/0/0/0/0	NA	NA
59		HIR	c.718G>T	E12	F	early child-hood	N/0/0/0/0	AChEIs-	-
60	[11]	LMR	g.393+1G>A	I5	M	Birth	0/0/0/0/0	AChEIs-/BA+++	+++
61		LMR	g.393+1G>A	I5	M	Birth	0/0/0/0/0	AChEIs-/BA+++	+++
62		LMR	g.393+1G>A	I5	F	EI	0/0/0/0/0	AChEIs-/BA+++	+++
63		LMR	g.393+1G>A	I5	M	Birth	0/0/0/0/0	AChEIs-/BA+++	+++
64		LMR	g.393+1G>A	I5	M	EI	0/0/0/0/0	AChEIs-/BA+++	+++
65		LMR	g.393+1G>A;g.718-1G>T	I5;I11	F	Birth	0/0/0/0/0	AChEIs-/BA+++	+++
66		LMR	g.393+1G>A;c.865G>T	I5;E13	F	Birth	0/0/0/0/0	AChEIs-/BA+++	+++
67	[37]	LMR	c.1082del	E15	F	Birth	0/N/0/Y/Y	BA++	++
68	[38]	LMR	NA	NA	M	4	Y/0/0/Y/N	BA++	++
69		LMR	NA	NA	M	12	Y/0/0/Y/N	AChEIs-/FLX-/BA++	++
70		LMR	NA	NA	M	7	N/0/0/N/Y	NA	NA
71		LMR	NA	NA	F	6.5	N/0/0/N/N	BA++	++
72	[30]	HIR	NA	NA	M	5	0/0/0/0/0	AChEIs-/BA+++	+++
73		HIR	c.1289A>C;g.1196G>A	E16;I15	F	Birth	0/0/0/0/0	AChEIs-/BA+++	+++
74	[10]	HIR	g.321-3T>G;g.393+1G>A	I3;I5	M	Birth	N/Y/N/0/Y	AChEIs-/BA+++	+++
75		HIR	g.321-3T>G;g.393+1G>A	I3;I5	M	Birth	N/Y/N/0/N	AChEIs-/BA++	++

76	[5]	HIR	c.176C>A	E2	NA	2	N/0/0/0/0	AChEIs-	-
77		HIR	c.1026C>G;c.1082del	E14;E15	NA	Birth	N/0/0/0/0	AChEIs-	-
78		HIR	c.375del;g.1195+1G>A	E5;I15	NA	Birth	N/0/0/0/0	AChEIs-	-
79		HIR	c.444G>A	E6	NA	Birth	Y/0/0/0/0	AChEIs-	-
80		HIR	c.805_806insC	E12	NA	first year	Y/0/0/0/0	AChEIs-	-
81		HIR	c.1111C>T;g.1195+1G>A	E15;I15	NA	Birth	N/0/0/0/0	AChEIs-	-
82		HIR	c.1111C>T;c.1219G>A;c.1331G>A	E15;E16;E17	NA	first year	N/0/0/0/0	AChEIs-	-
83	[32]	HIR	c.1321A>G	E17	M	8	N/Y/0/0/N	AChEIs-/DAP-	-
84		HIR	c.1321A>G	E17	F	3	N/Y/0/0/N	NA	NA
85		HIR	c.1321A>G	E17	F	EI	N/0/0/0/Y	AChEIs-/DAP+	+
86	[19]	HIR	c.943C>T;g.1300+3A>G	E13;I16	M	8	0/0/0/0/0	AChEIs-	-
87	[18]	LMR	c.1204C>T;c.1228C>T	E16	M	5	Y/0/0/0/0	AChEIs-/BA+++	+++
88		LMR	c.1228C>T	E16	M	3	Y/0/0/0/0	BA+++	+++
89		LMR	c.847G>T;c.1183G>A	E13;E15	M	0.5	N/0/0/0/0	AChEIs-	-
90		LMR	c.1228C>T	E16	M	9	Y/0/0/0/0	BA+++	+++
91		LMR	c.1228C>T	E16	F	12	Y/0/0/0/0	BA+++	+++
92	[15]	HIR	c.679C>T	E11	M	Birth	0/0/0/0/Y	AChEIs-/BA++	++
93	[39]	HIR	NA	NA	F	NA	0/0/0/Y/0	BA++	++
94	[14]	HIR	g.1195+1G>A;c.1289A>C	I15;E16	F	2m	0/0/0/N/0	BA+++	+++
95		HIR	c.157dup	E2	M	Birth	0/0/0/Y/0	BA+++	+++
96		HIR	c.1289A>C	E16	F	6	0/0/0/N/0	BA+++	+++
97		HIR	c.1289A>C	E16	F	7	0/0/0/N/0	BA+++	+++
98		HIR	c.1289A>C	E16	F	8	0/0/0/N/0	BA+++	+++
99		HIR	c.1289A>C	E16	M	<1	0/0/0/N/0	BA+++	+++
100		HIR	c.1289A>C	E16	F	5	0/0/0/N/0	BA+++	+++
101		HIR	c.1289A>C	E16	F	10	0/0/0/N/0	BA+++	+++
102		HIR	c.640G>T;c.1289A>C	E11;E16	F	18m	0/0/0/N/0	BA+++	+++
103	[26]	LMR	c.1169A>G	E15	NA	1.5	Y/0/N/0/0	BA+++	+++
104		LMR	c.444G>A	E6	NA	0.5	Y/0/N/0/Y	BA+++/DAP++	+++
105		LMR	c.444G>A	E6	NA	11m	Y/0/N/0/0	BA+++	+++
106		LMR	c.444G>A	E6	NA	0.6m	Y/0/N/0/0	BA+++	+++
107		LMR	c.444G>A	E6	NA	8m	Y/0/N/0/0	BA+++	+++
108	[40]	LMR	NA	NA	F	Birth	0/0/0/0/0	AChEIs-/BA++	++
109		LMR	NA	NA	M	Birth	0/0/0/0/0	AChEIs-/FLX++	++
110		LMR	NA	NA	F	Birth	0/0/0/0/0	AChEIs-/BA++/FLX++	++
111		LMR	NA	NA	M	Birth	0/0/0/0/0	AChEIs-	-
112		LMR	NA	NA	F	Birth	0/0/0/0/0	AChEIs-/BA++/FLX++	++
113	[41]	HIR	c.679C>T;c.1228C>T	E11;E16	M	6m	N/0/0/0/Y	AChEIs-/OB-/BA++	++
114	[8]	HIR	g.218+1G>A;g.1197-1G>A	I2;I15	M	EI	N/Y/0/0/0	BA++	++

115	[42]	HIR	NA	NA	NA	NA	0/0/0/0/0	BA++	++
116		HIR	NA	NA	NA	NA	0/0/0/0/0	AChEIs-	-
117	[12]	LMR	c.444G>A	E6	F	NA	0/0/0/0/Y	AChEIs-/BA+++	+++
118		LMR	c.444G>A	E6	F	NA	0/0/0/0/Y	AChEIs-/BA+++	+++
119		LMR	c.444G>A	E6	F	NA	0/0/0/0/Y	AChEIs-/BA+++	+++
120		LMR	c.444G>A	E6	F	NA	0/0/0/0/Y	AChEIs-/BA+++	+++
121		LMR	NA	NA	M	NA	0/0/0/0/Y	AChEIs-/BA+++	+++
122	[30]	HIR	NA	NA	NA	NA	0/0/0/0/0	AChEIs-	-
123		HIR	NA	NA	NA	NA	0/0/0/0/0	AChEIs-	-
124		HIR	NA	NA	NA	NA	0/0/0/0/0	BA++	++
125		HIR	NA	NA	NA	NA	0/0/0/0/0	BA++	++
126		HIR	NA	NA	NA	NA	0/0/0/0/0	BA++	++
127		HIR	NA	NA	NA	NA	0/0/0/0/0	BA++	++
128	[28]	LMR	c.1082del;c.1190G>A	E15	F	0-1	0/Y/0/Y/0	AChEIs-/BA+	+
129		LMR	c.444G>A	E6	M	2	0/N/0/Y/Y	AChEIs-	-
130	[6]	HIR	c.107_215del;c.640G>T	E2,3;E11	M	NA	0/0/0/0/0	AChEIs-	-
131		HIR	c.506C>G	E7	NA	NA	0/0/0/0/0	AChEIs-	-
132		HIR	c.844A>T;c.1082del	E13;E15	NA	NA	0/0/0/0/0	AChEIs-	-
133		HIR	c.787_788insC	E12	M	NA	0/0/0/0/0	AChEIs-/OD-/GCs+	+
134		HIR	c.1082del	E15	NA	NA	0/0/0/0/0	AChEIs-	-
135		HIR	c.640G>T	E11	NA	NA	0/0/0/0/0	AChEIs-	-
136	[31]	HIR	c.1289A>C	E16	M	NA	0/0/0/0/0	AChEIs-	-
137		HIR	c.1289A>C	E16	M	NA	0/0/0/0/0	AChEIs-	-
138		HIR	c.1289A>C	E16	M	NA	0/0/0/0/0	AChEIs-	-
139		HIR	c.1289A>C	E16	M	NA	0/0/0/0/0	AChEIs-	-
140		HIR	c.1289A>C	E16	F	NA	0/0/0/0/0	AChEIs-	-
141		HIR	c.1289A>C	E16	F	NA	0/0/0/0/0	AChEIs-	-
142	[22]	HIR	c.679C>T;c.965T>A	E11;E14	M	Birth	0/0/0/0/Y	AChEIs-	-
143		HIR	c.1339G>C	E17	M	Birth	0/0/0/0/Y	AChEIs-	-
144		HIR	c.1331G>A;c.1354C>T	E17	M	3	0/0/0/0/N	AChEIs++	++
145	[24]	HIR	c.631C>T;c.1199G>A	E10;E16	NA	Birth	N/0/0/0/0	AChEIs-	-
146		HIR	c.631C>T;c.1199G>A	E10;E16	NA	Birth	N/0/0/0/0	AChEIs-	-
147		HIR	c.1108G>A;c.1210T>G;c.1268G>T	E15;E16	NA	Birth	N/0/0/0/0	AChEIs-	-
148		HIR	c.1108G>A;c.1210T>G;c.1268G>T	E15;E16	NA	Birth	N/0/0/0/0	AChEIs-	-
149		HIR	c.1108G>A;c.1268G>T	E15;E16	NA	Birth	N/0/0/0/0	AChEIs-	-
150		HIR	c.1082del;p.C397S	E15	NA	Birth	N/0/0/0/Y	AChEIs-	-
151		HIR	c.1214G>T	E16	NA	Birth	N/0/0/0/Y	AChEIs-	-
152	[21]	LMR	g.955-2A>C	I13	NA	NA	0/0/0/0/0	BA++	++

153		LMR	g.955-2A>C	I13	NA	NA	0/0/0/0/0	BA++	++
154	[48]	HIR	NA	NA	M	childhood	0/0/0/0/0	BA+++	+++
155		HIR	NA	NA	M	2 years	0/0/0/0/0	BA+++	+++
156		HIR	NA	NA	F	NA	0/0/0/0/0	NA	NA
157	[1]	HIR	g.107-2A>G;c.1354C>T	I1;E17	M	3m	0/0/0/0/0	AChEIs-/BA+++/DAP+++	+++
158	[49]	LMR	NA	I12	M	10	N/Y/0/Y/0	AChEIs-	-
159	[50]	HIR	NA	NA	M	NA	0/0/0/0/0	AChEIs-/GCs+	+
160		HIR	NA	NA	F	NA	0/0/0/0/0	AChEIs-/GCs+	+
161		HIR	NA	NA	M	NA	0/0/0/0/0	AChEIs-/GCs+	++
162	[51]	LMR	g.219+1G>C;c.1019G>A	I2;E14	NA	NA	0/0/0/0/0	AChEIs+	+
163		LMR	g.219+1G>C;c.1019G>A	I2;E14	NA	NA	0/0/0/0/0	NA	NA
164		LMR	g.219+1G>C;c.1019G>A	I2;E14	NA	NA	0/0/0/0/0	NA	NA

In **Supplementary Table 3**, we listed the information of each patient. **GS** means geographical settings of each patient. **HIR** refers to high-income-regions; **LMR** refers to low-and middle-income regions. In the description of mutation position, **E** refers to exon and **I** refers to intron. Onset Age refers to the age when symptoms of CMS appear. **EI** refers to early infancy. **NA** refers to not available information.

IM/NPD/I/MD/V refers to intermarriage/normal pregnancy and delivery/experience of respiratory infections/motor function developmental delay/assisted ventilation. **Y** and **N** means yes and no. **0** means the information isn't mentioned.

In the line of treatment, the drug type and effect are listed. The drugs include acetylcholinesterase inhibitors **(AChEIs)**, β-adrenergic receptor agonists **(BAs)**, 3,4-diaminopyridine **(DAP)**, fluoxetine **(FLX)**, glucocorticosteroids **(GCs)** and other drugs. Other drug A **(OA)** refers to L-Carnitine and Coenzyme Q10. Other drug B **(OB)** refers to cocktail of carnitine, thiamine, riboflavin, menadione and Coenzyme Q. Other drug C **(OC)** refers to the combination of levetiracetam, carbamazepine, midazolam, clonazepam, vitamin B6, and vigabatrin.

Treatment effect is categorized into four types: **(-)** no effect, negative or ambiguous response; **(+)** partial, incomplete, moderate, modest/mild effect or denotes initial positive response followed by ineffectiveness; **(++)** beneficial, positive or clear effect; **(+++)** remarkable, dramatical or satisfying effect.

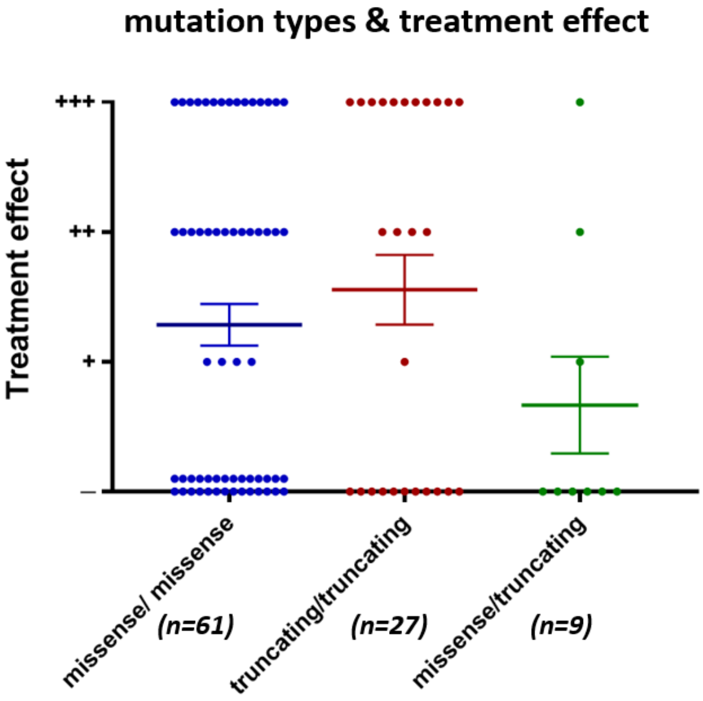

Supplementary Figure 1. Influence of Mutation Type on the Pharmacological Treatment Effects. The treatment effect was categorized into four types: (-) no effect, negative or ambiguous response; (+) partial, incomplete, moderate, modest/mild effect or denotes initial positive response followed by ineffectiveness and worsening; (++) beneficial, positive or clear effect; (+++) remarkable, dramatical or satisfying effect. Patients are divided in to different groups according to the *COLQ* mutation types: missense/missense, missense/truncating, truncating/truncating. Each point represents a patient. Mean and SEM are indicated. No significant difference was found by Kruskal-Wallis test ($p > 0.05$).

REFERENCES

[1] C. Anna, K.S. Yeon, L.J. Sook, L.B. Chan, K. Hunmin, H. Hee, C. Jong-Hee, Wide heterogeneity of congenital myasthenic syndromes: analysis of clinical experience in a tertiary center, Journal of Genetic Medicine, 17 (2020) 73-78.
[2] K. Ishigaki, D. Nicolle, E. Krejci, J.P. Leroy, J. Koenig, M. Fardeau, B. Eymard, D. Hantaï, Two novel mutations in the COLQ gene cause endplate acetylcholinesterase deficiency, Neuromuscul Disord, 13 (2003) 236-244.
[3] V. Mihaylova, J.S. Müller, J.J. Vilchez, M.A. Salih, M.M. Kabiraj, A. D'Amico, E. Bertini, J. Wölfle, F. Schreiner, G. Kurlemann, V.M. Rasic, D. Siskova, J. Colomer, A. Herczegfalvi, K. Fabriciova, B. Weschke, R. Scola, F. Hoellen, U. Schara, A. Abicht, H. Lochmüller, Clinical and molecular genetic findings in COLQ-mutant congenital myasthenic syndromes, Brain, 131 (2008) 747-759.
[4] I. Wargon, P. Richard, T. Kuntzer, D. Sternberg, S. Nafissi, K. Gaudon, A. Lebail, S. Bauche, D. Hantaï, E. Fournier, B. Eymard, T. Stojkovic, Long-term follow-up of patients with congenital myasthenic syndrome caused by COLQ mutations, Neuromuscul Disord, 22 (2012) 318-324.
[5] K. Ohno, A.G. Engel, J.M. Brengman, X.M. Shen, F. Heidenreich, A. Vincent, M. Milone, E. Tan, M. Demirci, P. Walsh, S. Nakano, I. Akiguchi, The spectrum of mutations causing end-plate acetylcholinesterase deficiency, Ann Neurol, 47 (2000) 162-170.
[6] K. Ohno, J. Brengman, A. Tsujino, A.G. Engel, Human endplate acetylcholinesterase deficiency caused by mutations in the collagen-like tail subunit (ColQ) of the asymmetric enzyme, Proc Natl Acad Sci U S A, 95 (1998) 9654-9659.
[7] C.G. Tay, C.Y. Fong, L. Li, V. Ganesan, C.M. Teh, C.S. Gan, M.K. Thong, Congenital myasthenic syndrome with novel pathogenic variants in the COLQ gene associated with the presence of antibodies to acetylcholine receptors, J Clin Neurosci, 72 (2020) 468-471.
[8] S.H. Chan, V.C. Wong, A.G. Engel, Neuromuscular junction acetylcholinesterase deficiency responsive to albuterol, Pediatr Neurol, 47 (2012) 137-140.
[9] Y.A. Shapira, M.E. Sadeh, M.P. Bergtraum, A. Tsujino, K. Ohno, X.M. Shen, J. Brengman, S. Edwardson, I. Matoth, A.G. Engel, Three novel COLQ mutations and variation of phenotypic expressivity due to G240X, Neurology, 58 (2002) 603-609.
[10] W.L. Yeung, C.W. Lam, P.C. Ng, Intra-familial variation in clinical manifestations and response to ephedrine in siblings with congenital myasthenic syndrome caused by novel COLQ mutations, Dev Med Child Neurol, 52 (2010) e243-244.
[11] N. Pattrakornkul, C. Ittiwut, P. Boonsimma, K. Boonyapisit, C. Khongkhatithum, O. Sanmaneechai, K. Suphapeetiporn, V. Shotelersuk, Congenital myasthenic syndromes in the Thai population: Clinical findings and novel mutations, Neuromuscul Disord, 30 (2020) 851-858.
[12] H. Durmus, X.M. Shen, P. Serdaroglu-Oflazer, B. Kara, Y. Parman-Gulsen, C. Ozdemir, J. Brengman, F. Deymeer, A.G. Engel, Congenital myasthenic syndromes in Turkey: Clinical clues and prognosis with long term follow-up, Neuromuscul Disord, 28 (2018) 315-322.
[13] J.J. Pallithanam, S.P. Prabhudesai, N. Naik, S. Gauns, COLQ-Related Congenital Myasthenic Syndrome in a Child from Western India, Neurol India, 69 (2021) 228-229.
[14] D. Natera-de Benito, A. Töpf, J.J. Vilchez, L. González-Quereda, J. Domínguez-Carral, J. Díaz-Manera, C. Ortez, M. Bestué, P. Gallano, M. Dusl, A. Abicht, J.S. Müller, J. Senderek, A. García-Ribes, N. Muelas, T. Evangelista, Y. Azuma, G. McMacken, A. Paipa Merchan, P.M. Rodríguez Cruz, A. Camacho, E. Jiménez, M.C. Miranda-Herrero, A. Santana-Artiles, O. García-Campos, R. Dominguez-Rubio, M. Olivé, J. Colomer, D. Beeson, H. Lochmüller, A. Nascimento, Molecular characterization of congenital myasthenic syndromes in Spain, Neuromuscul Disord, 27 (2017) 1087-1098.
[15] D.E. Prior, P.S. Ghosh, Congenital Myasthenic Syndrome From a Single Center: Phenotypic and Genotypic features, J Child Neurol, 36 (2021) 610-617.
[16] N. Laforgia, L. De Cosmo, O. Palumbo, C. Ranieri, M. Sesta, D. Capodiferro, A. Pantaleo, P. Iapicca, P. Lastella, M. Capozza, F. Schettini, N. Bukvic, R. Bagnulo, N. Resta, The First Case of Congenital Myasthenic Syndrome Caused by a Large Homozygous Deletion in the C-Terminal Region of COLQ (Collagen Like Tail Subunit of Asymmetric Acetylcholinesterase) Protein, Genes (Basel), 11 (2020).
[17] H. Padmanabha, A.G. Saini, N. Sankhyan, P. Singhi, COLQ-Related Congenital Myasthenic Syndrome and Response to Salbutamol Therapy, J Clin Neuromuscul Dis, 18 (2017) 162-163.
[18] P. Selvam, G. Arunachal, S. Danda, A. Chapla, A. Sivadasan, M. Alexander, M.M. Thomas, N.J. Thomas, Congenital Myasthenic Syndrome: Spectrum of Mutations in an Indian Cohort, J Clin Neuromuscul Dis, 20 (2018) 14-27.
[19] K. Ohno, J.M. Brengman, K.J. Felice, D.R. Cornblath, A.G. Engel, Congenital end-plate acetylcholinesterase deficiency caused by a nonsense mutation and an A-->G splice-donor-site mutation at position +3 of the collagenlike-tail-subunit gene (COLQ): how does G at position +3 result in aberrant splicing?, Am J Hum Genet, 65 (1999) 635-644.
[20] F. Schreiner, M. Hoppenz, R. Klaeren, J. Reimann, J. Woelfle, Novel COLQ mutation 950delC in synaptic congenital myasthenic syndrome and symptomatic heterozygous relatives, Neuromuscul Disord, 17 (2007) 262-265.
[21] S. Mishra, K. Girisha, A. Shukla, Further delineation of clinical and molecular characteristics of congenital myasthenic syndromes in Indian families, European Journal of Human Genetics, 28 (2020) 428-428.

[22] T. Nakata, M. Ito, Y. Azuma, K. Otsuka, Y. Noguchi, H. Komaki, A. Okumura, K. Shiraishi, A. Masuda, J. Natsume, S. Kojima, K. Ohno, Mutations in the C-terminal domain of ColQ in endplate acetylcholinesterase deficiency compromise ColQ-MuSK interaction, Hum Mutat, 34 (2013) 997-1004.

[23] H.N. Matlik, R.M. Milhem, I.Y. Saadeldin, H.S. Al-Jaibeji, L. Al-Gazali, B.R. Ali, Clinical and molecular analysis of a novel COLQ missense mutation causing congenital myasthenic syndrome in a Syrian family, Pediatr Neurol, 51 (2014) 165-169.

[24] J. Arredondo, M. Lara, F. Ng, D.A. Gochez, D.C. Lee, S.P. Logia, J. Nguyen, R.A. Maselli, COOH-terminal collagen Q (COLQ) mutants causing human deficiency of endplate acetylcholinesterase impair the interaction of ColQ with proteins of the basal lamina, Hum Genet, 133 (2014) 599-616.

[25] X. Luo, C. Wang, L. Lin, F. Yuan, S. Wang, Y. Wang, A. Wang, C. Wang, S. Wu, X. Lan, Q. Xu, R. Yin, H. Cheng, Y. Zhang, J. Xi, J. Zhang, X. Sun, J. Yan, F. Zeng, Y. Chen, Mechanisms of Congenital Myasthenia Caused by Three Mutations in the COLQ Gene, Front Pediatr, 9 (2021) 679342.

[26] G. Gül Mert, N. Özcan, Ö. Hergüner, Ş. Altunbaşak, F. Incecik, A. Bişgin, S. Ceylaner, Congenital myasthenic syndrome in Turkey: clinical and genetic features in the long-term follow-up of patients, Acta Neurol Belg, 121 (2021) 529-534.

[27] R. Al-Shahoumi, L.I. Brady, J. Schwartzentruber, M.A. Tarnopolsky, Two cases of congenital myasthenic syndrome with vocal cord paralysis, Neurology, 84 (2015) 1281-1282.

[28] Q. Ding, D. Shen, Y. Dai, Y. Hu, Y. Guan, M. Liu, L. Cui, Mechanism hypotheses for the electrophysiological manifestations of two cases of endplate acetylcholinesterase deficiency related congenital myasthenic syndrome, J Clin Neurosci, 48 (2018) 229-232.

[29] B. Gandolfi, R.A. Grahn, E.K. Creighton, D.C. Williams, P.J. Dickinson, B.K. Sturges, L.T. Guo, G.D. Shelton, P.A. Leegwater, M. Longeri, R. Malik, L.A. Lyons, COLQ variant associated with Devon Rex and Sphynx feline hereditary myopathy, Anim Genet, 46 (2015) 711-715.

[30] M. Bestue-Cardiel, A. Sáenz de Cabezón-Alvarez, J.L. Capablo-Liesa, J. López-Pisón, J.L. Peña-Segura, J. Martin-Martinez, A.G. Engel, Congenital endplate acetylcholinesterase deficiency responsive to ephedrine, Neurology, 65 (2005) 144-146.

[31] C. Donger, E. Krejci, A.P. Serradell, B. Eymard, S. Bon, S. Nicole, D. Chateau, F. Gary, M. Fardeau, J. Massoulié, P. Guicheney, Mutation in the human acetylcholinesterase-associated collagen gene, COLQ, is responsible for congenital myasthenic syndrome with end-plate acetylcholinesterase deficiency (Type Ic), Am J Hum Genet, 63 (1998) 967-975.

[32] J.S. Müller, S. Petrova, R. Kiefer, R. Stucka, C. König, S.K. Baumeister, A. Huebner, H. Lochmüller, A. Abicht, Synaptic congenital myasthenic syndrome in three patients due to a novel missense mutation (T441A) of the COLQ gene, Neuropediatrics, 35 (2004) 183-189.

[33] J.T. den Dunnen, R. Dalgleish, D.R. Maglott, R.K. Hart, M.S. Greenblatt, J. McGowan-Jordan, A.F. Roux, T. Smith, S.E. Antonarakis, P.E. Taschner, HGVS Recommendations for the Description of Sequence Variants: 2016 Update, Hum Mutat, 37 (2016) 564-569.

[34] M.A. Al-Muhaizea, S.B. Al-Mobarak, COLQ-mutant Congenital Myasthenic Syndrome with Microcephaly: A Unique Case with Literature Review, Transl Neurosci, 8 (2017) 65-69.

[35] A. Guven, M. Demirci, B. Anlar, Recurrent COLQ mutation in congenital myasthenic syndrome, Pediatr Neurol, 46 (2012) 253-256.

[36] A. Vidanagamage, I.K. Gooneratne, S. Nandasiri, K. Gunaratne, A. Fernando, S. Maxwell, J. Cossins, D. Beeson, T. Chang, A rare mutation in the COLQ gene causing congenital myasthenic syndrome with remarkable improvement to fluoxetine: A case report, Neuromuscul Disord, 31 (2021) 246-248.

[37] G.S. Duran, T.A. Uzunhan, B. Ekici, A. Çıtak, N. Aydınlı, M. Çalışkan, Severe scoliosis in a patient with COLQ mutation and congenital myasthenic syndrome: a clue for diagnosis, Acta Neurol Belg, 113 (2013) 531-532.

[38] V. Wadwekar, S.S. Nair, V. Tandon, A. Kuruvilla, M. Nair, Congenital myasthenic syndrome: Ten years clinical experience from a quaternary care south-Indian hospital, J Clin Neurosci, 72 (2020) 238-243.

[39] S. Caggiano, S. Khirani, E. Verrillo, C. Barnerias, A. Amaddeo, C. Gitiaux, B. Thierry, I. Desguerre, R. Cutrera, B. Fauroux, Sleep in infants with congenital myasthenic syndromes, Eur J Paediatr Neurol, 21 (2017) 842-851.

[40] Y. Zhao, Y. Li, Y. Bian, S. Yao, P. Liu, M. Yu, W. Zhang, Z. Wang, Y. Yuan, Congenital myasthenic syndrome in China: genetic and myopathological characterization, Ann Clin Transl Neurol, 8 (2021) 898-907.

[41] Y. Guo, M.J. Menezes, M.P. Menezes, J. Liang, D. Li, L.G. Riley, N.F. Clarke, P.I. Andrews, L. Tian, R. Webster, F. Wang, X. Liu, Y. Shen, D.R. Thorburn, B.J. Keating, A. Engel, H. Hakonarson, J. Christodoulou, X. Xu, Delayed diagnosis of congenital myasthenia due to associated mitochondrial enzyme defect, Neuromuscul Disord, 25 (2015) 257-261.

[42] J.C. Kao, M. Milone, D. Selcen, X.M. Shen, A.G. Engel, T. Liewluck, Congenital myasthenic syndromes in adult neurology clinic: A long road to diagnosis and therapy, Neurology, 91 (2018) e1770-e1777.

[43] U. Yiş, K. Becker, S.H. Kurul, G. Uyanik, E. Bayram, G. Haliloğlu, A. Polat, M. Ayanoğlu, D. Okur, A.F. Tosun, G. Serdaroğlu, S. Yilmaz, H. Topaloğlu, B. Anlar, S. Cirak, A.G. Engel, Genetic Landscape of Congenital Myasthenic Syndromes From Turkey: Novel Mutations and Clinical Insights, Journal of child neurology, 32 (2017) 759-765.

[44] F. Albassam, A. Alsaman, COLQ Mutation in Three Siblings of a Saudi Family: Congenital Myasthenia with Phenotypic Heterogeneity and Varying Ages of Onset, Neurology, 86 (2016) 2.

[45] D. Joshi, S. Patil, P. Dash, V. Mishra, R. Chaurasia, A. Pathak, Congenital myathenic syndrome associated with COLQ mutation: an interesting report, Journal of the neurological sciences, 405 (2019) 1.
[46] D.O. Hutchinson, T.J. Walls, S. Nakano, S. Camp, P. Taylor, C.M. Harper, R.V. Groover, H.A. Peterson, D.G. Jamieson, A.G. Engel, Congenital endplate acetylcholinesterase deficiency, Brain : a journal of neurology, 116 (Pt 3) (1993) 633-653.
[47] E.P. Estephan, A.A. Zambon, R. Thompson, K. Polavarapu, D. Jomaa, A. Töpf, P.V.P. Helito, C.O. Heise, C.A.M. Moreno, A.M.S. Silva, J.A. Kouyoumdjian, M.D.P. Morita, U.C. Reed, H. Lochmüller, E. Zanoteli, Congenital myasthenic syndrome: Correlation between clinical features and molecular diagnosis, European journal of neurology, (2021).
[48] F. Albassam, A. Alsaman, COLQ Mutation in Three Siblings of a Saudi Family: Congenital Myasthenia with Phenotypic Heterogeneity and Varying Ages of Onset, Neurology, 86 (2016).
[49] D. Joshi, S. Patil, P. Dash, V. Mishra, R. Chaurasia, A. Pathak, Congenital myathenic syndrome associated with COLQ mutation: an interesting report, Journal of the Neurological Sciences, 405 (2019).
[50] D.O. Hutchinson, T.J. Walls, S. Nakano, S. Camp, P. Taylor, C.M. Harper, R.V. Groover, H.A. Peterson, D.G. Jamieson, A.G. Engel, Congenital endplate acetylcholinesterase deficiency, Brain : a journal of neurology, 116 (Pt 3) (1993) 633-653.
[51] E.P. Estephan, A.A. Zambon, R. Thompson, K. Polavarapu, D. Jomaa, A. Töpf, P.V.P. Helito, C.O. Heise, C.A.M. Moreno, A.M.S. Silva, J.A. Kouyoumdjian, M.D.P. Morita, U.C. Reed, H. Lochmüller, E. Zanoteli, Congenital myasthenic syndrome: Correlation between clinical features and molecular diagnosis, Eur J Neurol, 29 (2022) 833-842.

PRISMA 2020 Checklist

Section and Topic	Item #	Checklist item	Location where item is reported
**TITLE**			
Title	1	Pharmacological Treatments for Congenital Myasthenic Syndromes Caused by COLQ Mutations	Page 1
**ABSTRACT**			
Abstract	2	Background: Congenital myasthenic syndromes (CMS) refer to a series of inherited disorders caused by defects in various proteins. Mutation in the collagen-like tail subunit of asymmetric acetylcholinesterase (COLQ) is the second-most common cause of CMS. However, data on pharmacological treatments are limited.<br>Objective: In this study, we reviewed related reports to determine the most appropriate pharmacological strategy for CMS caused by COLQ mutations. A literature review and meta-analysis were also performed. PubMed, MEDLINE, Web of Science, and Cochrane Library databases were searched to identify studies published in English before July 22, 2022.<br>Results: A total of 42 studies including 164 patients with CMS due to 72 different COLQ mutations were selected for evaluation. Most studies were case reports, and none were randomized clinical trials. Our meta-analysis revealed evidence that β-adrenergic agonists, including salbutamol and ephedrine, can be used as first-line pharmacological treatments for CMS patients with COLQ mutations, as 98.7% of patients (74/75) treated with β-adrenergic agonists showed positive effects. In addition, AChEIs should be avoided in CMS patients with COLQ mutations, as 90.5% (105/116) of patients treated with AChEIs showed either no or negative effects.<br>Conclusion: (1) β-adrenergic agonist therapy is the first pharmacological strategy for treating CMS with COLQ mutations. (2) AChEIs should be avoided in patients with CMS with COLQ mutations.	Page 1
**INTRODUCTION**			
Rationale	3	Different therapies have been applied for treating CMS in clinical settings. However, due to the interactions of various mechanisms, the same drug may have varying or even opposite effects on different CMS subtypes, which makes it necessary to analyze pharmacological treatments based on disease subtypes. Here, we reviewed related reports and attempted to determine the most appropriate pharmacological strategy for CMS caused by COLQ mutations.	Page 3
Objectives	4	To determine the most appropriate pharmacological strategy for CMS caused by COLQ mutations.	Page 3
**METHODS**			
Eligibility criteria	5	Case reports and studies were included if they fulfilled the following criteria: (1) English-language articles; (2) patients with COLQ mutations with no restriction regarding their age, sex, ethnicity, and treatment; and (3) genetic tests confirmed the COLQ mutation regardless of the details of the mutations. The exclusion criteria were as follows: (1) articles not reporting pharmacological treatment(s) and (2) articles that did not mention the treatment effect of a pharmacological therapy.	Page 3
Information sources	6	The literature search was restricted to articles published in English. We searched the PubMed (1966-2022), MEDLINE (1950-2022), Web of Science (1864-2022), and Cochrane Library (2022) databases. The most recent search was performed on July 22, 2022. In addition, a manual search was performed to identify references in the obtained studies to identify other possible studies.	Page 3
Search strategy	7	The keywords "COLQ mutations," "COLQ," or "congenital myasthenic syndrome, COLQ" were used.	Page 3
Selection process	8	The studies were read thoroughly to assess their eligibility for inclusion in the meta-analysis. SS and GS extracted data and performed the analysis.	Page 3,8
Data collection process	9	Because the effects of different strategies are described as negative, partial, positive, or remarkable, we categorized the treatment effect into four types: (-) no effect, negative or ambiguous response; (+) partial, incomplete, moderate, modest/mild effect, or initial positive response followed by ineffectiveness; (++) beneficial, positive, or clear effect; (+++) remarkable, dramatic, or satisfying effect.	Page 4
Data items	10a	Treatment effects: (-) no effect, negative or ambiguous response; (+) partial, incomplete, moderate, modest/mild effect, or initial positive response followed by ineffectiveness; (++) beneficial, positive, or clear effect; (+++) remarkable, dramatic, or satisfying effect.	Page 4
	10b	The following data were extracted directly from the articles: clinical information, gene mutations, pharmacological treatments, and follow-up data. Clinical data including sex, age at symptom onset, and age at the time of the case study were reported. If more than one patient had been reported in different studies or by the same group, the participants in the studies were compared. Data that appeared more than once were excluded from the analysis.	Page 3
Study risk of bias assessment	11	The quality of individual studies was assessed using the recently published "Tool for evaluating the methodological quality of case reports and case series" proposed by Murad et al. [36] Based on the previous criteria of the Pierson, Bradford Hills, and Newcastle-Ottawa scale. Each study was independently evalua-	Page 3

Section and Topic	Item #	Checklist item	Location where item is reported
		ted according to four domains (selection, ascertainment, causality, and reporting) to yield an overall assessment (Supplementary Table S2). The quality assessment included eight leading exploratory questions with a binary response (yes/no) to determine whether the items suggested the presence of bias.	
Effect measures	12	Data analysis was performed using Statistical Package for Social Sciences (SPSS Inc., Chicago, IL, USA) version 25. Statistical differences were estimated using the Kruskal-Wallis test or one-way analysis of variance followed by the Bonferroni post-hoc test. The analysis of the relationship between sex and treatment effect was estimated using the Mann-Whitney test (two groups). Simple linear regression was used to analyze the relationship between age of onset and treatment effect. Statistical significance was set at $p < 0.05$.	Page 3
Synthesis methods	13a	The following data were extracted directly from the articles: clinical information, gene mutations, pharmacological treatments, and follow-up data. Clinical data including sex, age at symptom onset, and age at the time of the case study were reported. If more than one patient had been reported in different studies or by the same group, the participants in the studies were compared. Data that appeared more than once were excluded from the analysis.	Page 3
	13b	The quality of individual studies was assessed using the recently published "Tool for evaluating the methodological quality of case reports and case series" proposed by Murad et al. [36] Based on the previous criteria of the Pierson, Bradford Hills, and Newcastle-Ottawa scale. Each study was independently evaluated according to four domains (selection, ascertainment, causality, and reporting) to yield an overall assessment (Supplementary Table S2). The quality assessment included eight leading exploratory questions with a binary response (yes/no) to determine whether the items suggested the presence of bias.	Page 3
	13c	Statistical differences were estimated using the Kruskal-Wallis test or one-way analysis of variance followed by the Bonferroni post-hoc test. The analysis of the relationship between sex and treatment effect was estimated using the Mann-Whitney test (two groups). Simple linear regression was used to analyze the relationship between age of onset and treatment effect. Statistical significance was set at $p < 0.05$.	Page 3
	13d	Data analysis was performed using Statistical Package for Social Sciences (SPSS Inc., Chicago, IL, USA) version 25.	Page 3
	13e	Statistical differences were estimated using the Kruskal-Wallis test or one-way analysis of variance followed by the Bonferroni post-hoc test. The analysis of the relationship between sex and treatment effect was estimated using the Mann-Whitney test (two groups). Simple linear regression was used to analyze the relationship between age of onset and treatment effect. Statistical significance was set at $p < 0.05$.	Page 3
	13f	Statistical differences were estimated using the Kruskal-Wallis test or one-way analysis of variance followed by the Bonferroni post-hoc test. The analysis of the relationship between sex and treatment effect was estimated using the Mann-Whitney test (two groups). Simple linear regression was used to analyze the relationship between age of onset and treatment effect. Statistical significance was set at $p < 0.05$.	Page 3
Reporting bias assessment	14	Each study was independently evaluated according to four domains (selection, ascertainment, causality, and reporting) to yield an overall assessment (Supplementary Table S2).	Page 3
Certainty assessment	15	The studies were read thoroughly to assess their eligibility for inclusion in the meta-analysis. SS and GS extracted data and performed the analysis.	Page 3,8
**RESULTS**			
Study selection	16a	After searching, 42 studies meeting our inclusion and exclusion criteria were selected, including 164 patients with CMS due to 72 different COLQ mutations [6, 12-14, 17, 19, 22, 27-30, 37-61]. Most selected studies were case reports, and none were RCTs. The information for each patient is listed in Supplementary Table S3, including geographical settings, mutation, onset age, related risk factors, treatment, and effects.	Page 4
	16b	All the studies matching our including criteria and not matching our excluding criteria were included.	Page 4
Study characteristics	17	Each study was independently evaluated according to four domains (selection, ascertainment, causality, and reporting) to yield an overall assessment (Supplementary Table S2).	Page 4
Risk of bias in studies	18	After searching, 42 studies meeting our inclusion and exclusion criteria were selected, including 164 patients with CMS due to 72 different COLQ mutations [6, 12-14, 17, 19, 22, 27-30, 37-61]. Most selected studies were case reports, and none were RCTs. The information for each patient is listed in Supplementary Table S3, including geographical settings, mutation, onset age, related risk factors, treatment, and effects.	Page 4
Results of individual studies	19	Six different pharmacological strategies were used, including AChE inhibitors (AChEIs), a type of drug that inhibits ACh hydrolysis; β-adrenergic receptor agonists (BAs); 3,4-DAP, a drug that increases ACh release from the nerve terminal by blocking voltage-gated potassium channels (VGKCs); fluoxetine (FLX); glucocorticosteroids (GCs); and other drugs (Table 1). Among the five different strategies, β-adrenergic receptor agonists showed better effects than 3,4-DAP, AChEIs, and glucocorticosteroids (Fig. 4).	Page 3,4
Results of syntheses	20a	Among the five different strategies, β-adrenergic receptor agonists showed better effects than 3,4-DAP, AChEIs, and glucocorticosteroids (Fig. 4).	Page 4
	20b	A total of 42 studies including 164 patients with CMS due to 72 different COLQ mutations were selected for	Page 4

Section and Topic	Item #	Checklist item	Location where item is reported
		evaluation. Most studies were case reports, and none were randomized clinical trials. Our meta-analysis revealed evidence that β-adrenergic agonists, including salbutamol and ephedrine, can be used as first-line pharmacological treatments for CMS patients with COLQ mutations, as 98.7% of patients (74/75) treated with β-adrenergic agonists showed positive effects. In addition, AChEIs should be avoided in CMS patients with COLQ mutations, as 90.5% (105/116) of patients treated with AChEIs showed either no or negative effects.	
	20c	However, there were some limitations to our meta-analysis. (1) There might have been publication bias due to the possibility that only positive results were published. (2) Most reports that we reviewed lacked aquantitative assessment of treatment effectiveness. Instead, they were described qualitatively using words such as negative, partial, and positive. This may also lead to bias and flatten variations in reported treatment outcomes. (3) Insufficient information was provided regarding drug doses, so it was difficult to discern dose-effect relationships for the investigated treatments.	Page 4,5
	20d	Forest plot was not made due to the special method used to analysis.	Page 5
Reporting biases	21	However, there were some limitations to our meta-analysis. (1) There might have been publication bias due to the possibility that only positive results were published. (2) Most reports that we reviewed lacked aquantitative assessment of treatment effectiveness. Instead, they were described qualitatively using words such as negative, partial, and positive. This may also lead to bias and flatten variations in reported treatment outcomes. (3) Insufficient information was provided regarding drug doses, so it was difficult to discern dose-effect relationships for the investigated treatments.	Page 5
Certainty of evidence	22	The β-adrenergic receptor agonists showed the best treatment effect, benefiting 74 of the 75 patients treated (98.7%) (Fig. 4). In addition, AChEIs showed no effect on 105 of the 116 patients treated (90.5%) (Fig. 4).	Page 5
**DISCUSSION**			
Discussion	23a	In our study, β-adrenergic receptor agonists performed best among all pharmacological methods for treating patients with CMS due to COLQ mutations. These drugs are widely used in clinical practice. The beneficial effect of ephedrine inpatients with myasthenia gravis has been recognized since the 1930s [70]. Physicians reported its effect on improvingmuscle strength and decreasing fatigability when treating dysmenorrhea by chance [71, 72], but anticholinesterases and corticosteroids have since replaced it owing to possible adverse effects [73]. Ephedrine returned to the spotlight again owing to its effect on CMS treatment, especially CMS caused by DOK7 mutations. DOK7 is a muscle-intrinsic activator of MuSK. Mutations in DOK7 may lead to DOK7 deficiency, thus leading to NMJ defects [3, 74, 75]. Although β-adrenergic receptor agonists have been proven effective in different subtypes of CMS, the exact mechanism remains unclear. β-adrenergic receptor agonists have been proven to be effective in most CMS patients with mutations such as SLC5A7, LAMB2, COL13A1, CHRNE, DOK7, and MUSK[32, 66, 76], but they have no effect in CMS patients with SYT2 [77], TOR1AIP1 [78], and CHD8 [79] mutations.	Page 8
	23b	However, there were some limitations to our meta-analysis. (1) There might have been publication bias due to the possibility that only positive results were published. (2) Most reports that we reviewed lacked aquantitative assessment of treatment effectiveness. Instead, they were described qualitatively using words such as negative, partial, and positive. This may also lead to bias and flatten variations in reported treatment outcomes. (3) Insufficient information was provided regarding drug doses, so it was difficult to discern dose-effect relationships for the investigated treatments.	Page 8
	23c	However, there were some limitations to our meta-analysis. (1) There might have been publication bias due to the possibility that only positive results were published. (2) Most reports that we reviewed lacked aquantitative assessment of treatment effectiveness. Instead, they were described qualitatively using words such as negative, partial, and positive. This may also lead to bias and flatten variations in reported treatment outcomes. (3) Insufficient information was provided regarding drug doses, so it was difficult to discern dose-effect relationships for the investigated treatments.	Page 8
	23d	However, there were some limitations to our meta-analysis. (1) There might have been publication bias due to the possibility that only positive results were published. (2) Most reports that we reviewed lacked aquantitative assessment of treatment effectiveness. Instead, they were described qualitatively using words such as negative, partial, and positive. This may also lead to bias and flatten variations in reported treatment outcomes. (3) Insufficient information was provided regarding drug doses, so it was difficult to discern dose-effect relationships for the investigated treatments.	Page 8
**OTHER INFORMATION**			
Registration and protocol	24a	This systematic review has not been registered in the international prospective register of systematic reviews (PROSPERO).	Page 8
	24b	This systematic review and meta-analysis protocol has not been published.	Page 8
	24c	This systematic review has not been registered in the international prospective register of systematic reviews (PROSPERO).	Page 8
Support	25	This work was supported by the Science and Technology Innovation Program of Hunan Province, China (Grant No. 2021RC2023, KH), the China Postdoctoral Science Foundation (Grant No. 2021M703638, KH), and the National Natural Science Foundation of China (Grant No. 82201557, KH).	Page 8

Section and Topic	Item #	Checklist item	Location where item is reported
Competing interests	26	The authors declare no conflict of interest, financial or otherwise.	Page 9
Availability of data, code and other materials	27	The information for each patient is listed in Supplementary Table S3, including geographical settings, mutation, onset age, related risk factors, treatment, and effects.	Page 9

From: Page MJ, McKenzie JE, Bossuyt PM, Boutron I, Hoffmann TC, Mulrow CD, et al. The PRISMA 2020 statement: an updated guideline for reporting systematic reviews. BMJ 2021;372:n71. doi: 10.1136/bmj.n71

For more information, visit: http://www.prisma-statement.org/
